# Targeting activin receptor–like kinase 7 ameliorates adiposity and associated metabolic disorders

**DOI:** 10.1172/jci.insight.161229

**Published:** 2023-02-22

**Authors:** Min Zhao, Katsuhide Okunishi, Yun Bu, Osamu Kikuchi, Hao Wang, Tadahiro Kitamura, Tetsuro Izumi

**Affiliations:** 1 Laboratory of Molecular Endocrinology and Metabolism, Department of Molecular Medicine, and; 2 Metabolic Signal Research Center, Institute for Molecular and Cellular Regulation, Gunma University, Maebashi, Japan.

**Keywords:** Metabolism, Adipose tissue, Cytokines

## Abstract

Activin receptor-like kinase 7 (ALK7) is a type I receptor in the TGF-β superfamily preferentially expressed in adipose tissue and associated with lipid metabolism. Inactivation of ALK7 signaling in mice results in increased lipolysis and resistance to both genetic and diet-induced obesity. Human genetic studies have recently revealed an association between ALK7 variants and both reduced waist to hip ratios and resistance to development of diabetes. In the present study, treatment with a neutralizing mAb against ALK7 caused a substantial loss of adipose mass and improved glucose intolerance and insulin resistance in both genetic and diet-induced mouse obesity models. The enhanced lipolysis increased fatty acid supply from adipocytes to promote fatty acid oxidation in muscle and oxygen consumption at the whole-body level. The treatment temporarily increased hepatic triglyceride levels, which resolved with long-term Ab treatment. Blocking of ALK7 signals also decreased production of its ligand, growth differentiation factor 3, by downregulating S100A8/A9 release from adipocytes and, subsequently, IL-1β release from adipose tissue macrophages. These findings support the feasibility of potential therapeutics targeting ALK7 as a treatment for obesity and diabetes.

## Introduction

Obesity due to the accumulation of excess fat as energy storage is a modern pandemic associated with significant comorbidities, such as type 2 diabetes, cardiovascular disease, arthritis, susceptibility to viral infections, and cancer. Interventions beyond changes in diet and exercise must be considered, given the complexity of the pathogenesis. Current therapeutic agents for obesity that mainly target appetite are not sufficiently effective (1). Although bariatric surgery, comprising excision or shunting of the upper gastrointestinal tract, remains the most common and effective therapy for extreme obesity, there remains an urgent need for less invasive approaches. To reduce accumulated fat nonsurgically, the lipids stored in adipocytes must first undergo lipolysis prior to their consumption in peripheral tissues. Thus, intervention in lipid metabolism in white adipocytes could show some promise for combating obesity regardless of its etiology.

Activin receptor-like kinase 7 (ALK7) is known to be predominantly expressed in adipose tissue compared with other tissues in both humans and mice (2, 3). Whole-body ALK7-KO mice show reduced fat accumulation when fed a high-fat diet (HFD) (4). Furthermore, its functional loss due to natural mutation incorporated into genetically determined obese and diabetic Tsumura, Suzuki, Obese Diabetes mice reduces fat mass by increasing lipolysis in adipocytes through elevated expression of adipose lipases (5). Furthermore, fat-specific ALK7-KO mice attenuate fat accumulation under an HFD potentially by increasing catecholamine (CA) sensitivity (6). ALK7 mediates signaling in adipocytes by its cognate ligand, growth differentiation factor 3 (GDF3), which is produced by adipose tissue macrophages (ATMs) and is upregulated by circulating insulin secreted under conditions of nutrient excess (7). Through suppression of lipolysis in adipocytes, this insulin/GDF3/ALK7 signaling axis is thought to play an important physiological role in storing excess caloric intake as fat. However, persistent activation of this axis can enlarge adipocytes, produce proinflammatory adipokines, and lead to insulin resistance (8). In fact, GDF3 is induced by NLRP3 inflammasome activation (9), which is primed by cytokines such as TNF-α or IL-1β in white adipose tissue (WAT) of obese patients (10). Consistent with findings that ALK7 or GDF3 deficiency inhibits accumulation of WAT in rodent models of diet-induced and genetic obesity (4, 5, 11), it has recently been reported that genetic variants of *ACVR1C* encoding ALK7 in humans are associated with reduced waist to hip ratios and protection against development of type 2 diabetes (12).

In the present study, we administrated a neutralizing mAb that binds the extracellular domain of ALK7 to genetically determined and diet-induced mouse models of obesity. We found that the anti-ALK7 treatment markedly reduces adiposity and markedly ameliorates obesity-associated metabolic disorders, thus implicating ALK7 as a promising target for obesity therapy. We further showed that blockade of ALK7 signals in adipocytes decreases production of GDF3 in ATMs by downregulating the release of S100A8/A9 from adipocytes and the resultant activation of NLRP3 and IL-1β in ATMs.

## Results

### ALK7-neutralizing Ab reduces adiposity in mouse models of genetic and dietary obesity.

Previous mouse and human genetic findings (5, 12) have identified ALK7-neutralizing Abs as potential therapeutics for obesity and diabetes. We obtained a human mAb against the human ALK7 extracellular domain that contains a murine IgG2a-Fc domain (ALK7 mAb). The mouse ALK7 extracellular domain exhibits greater than 97% aa sequence identity with its human counterpart. We first evaluated activity of the ALK7 mAb in a mouse model of genetic obesity, TSOD (13, 14).

The male mice were treated with ALK7 mAb, mouse IgG2a isotype Ab, or PBS twice weekly for 6 weeks from 5 to 11 weeks of age. CT scanning revealed significant decreases in adiposity at 10 and 15 mg/kg BW, but not at 3 mg/kg, after 3 weeks of treatment, and at all doses after 6 weeks of treatment (Supplemental Figure 1A; supplemental material available online with this article; https://doi.org/10.1172/jci.insight.161229DS1). In contrast, there were no changes in adiposity by treatment with 10 mg/kg isotype Ab or PBS. We hereafter administered 10 mg/kg of the Ab and PBS to male mice.

Consistent with the tissue distributions of ALK7 transcripts, the Ab was distributed markedly in epididymal WAT (epiWAT), but not significantly, in hypothalamus that was reported to express ALK7 (15) or liver that does not express it (Supplemental Figure 1B). The Ab injected only 1 time accumulated in epiWAT and upregulated the protein level of adipose triglyceride lipase (ATGL) maximally on days 2 and 3 (Supplemental Figure 1C). It did not increase the activity of the cytotoxic marker serum lactate dehydrogenase (LDH) (Supplemental Figure 1D).

**Figure 1:**
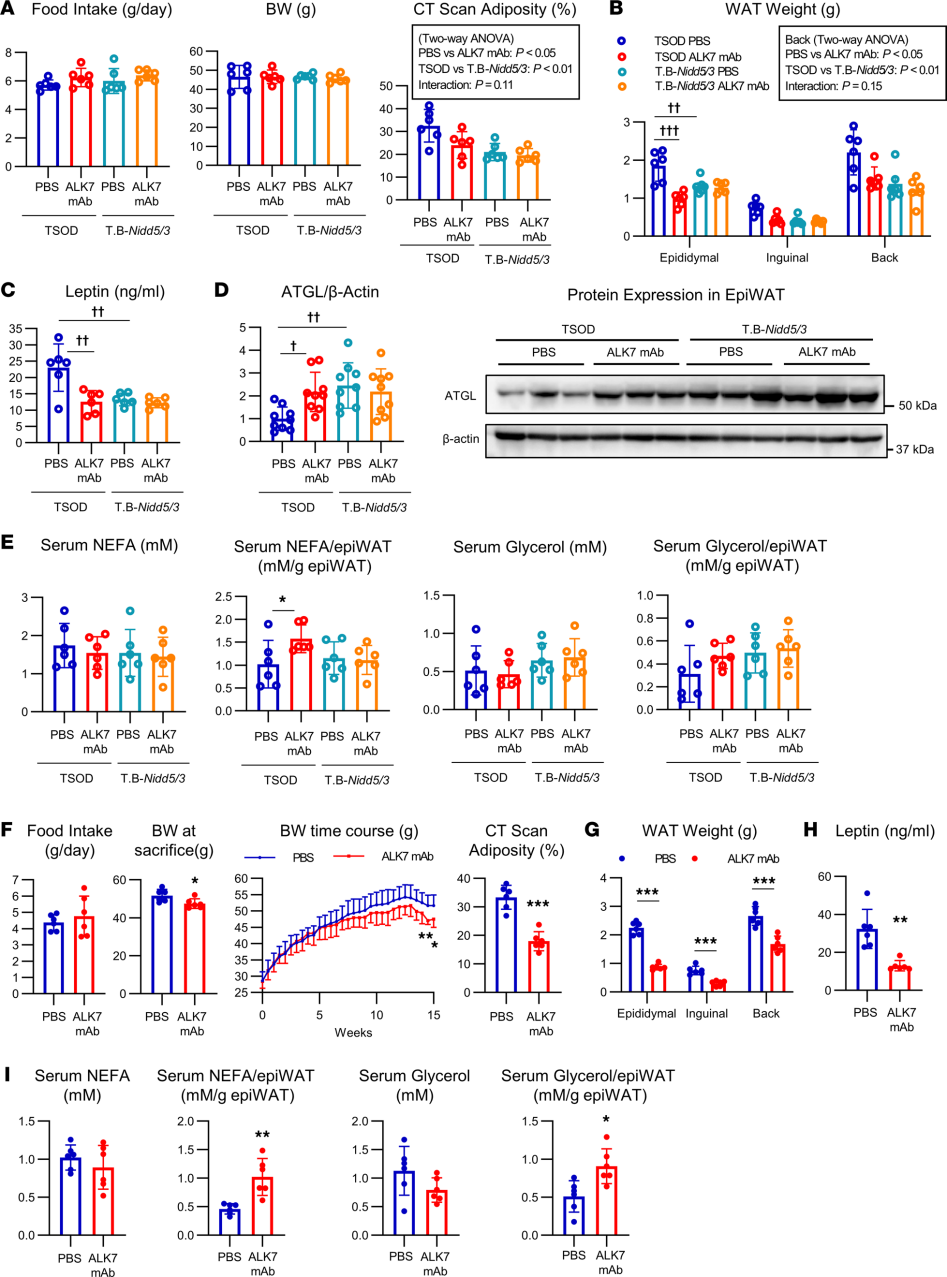
ALK7 mAb treatment reduces adiposity in ALK7-intact TSOD mice but not in ALK7-deficient counterparts. TSOD mice and their ALK7-deficient counterparts, T.B-*Nidd5*/3 mice, were treated with ALK7 mAb (10 mg/kg) or PBS from 5 to 11 weeks of age (**A**–**E**). Similarly, TSOD mice were treated with ALK7 mAb or PBS from 5 to 20 weeks of age (**F**–**I**). Measurements were made of food intake, BW, adiposity as determined by CT (**A** and **F**), fat pad weight (**B** and **G**), serum leptin concentration (**C** and **H**), ATGL protein expression levels in epiWAT (**D**), and serum concentrations of NEFA and glycerol and those normalized by weights of epiWAT (**E** and **I**). Phenotypic measurement and sample preparation were performed at the end of each cohort, except that food intake and CT were analyzed at 1 week before killing the mice (*n* = 6 each). The quantification of the ATGL and β-actin protein levels was based on densitometric analyses of immunoblots (*n* = 9). A representative blot of epiWAT extracts (15 μg of protein) from 3 mice per group is shown (**D**). ^†^*P* < 0.05, ^††^*P* < 0.01, ^†††^*P* < 0.001 by 2-way ANOVA followed by Tukey multiple comparison. *P < 0.05, **P < 0.01, ***P < 0.001 by *t* test.

We then compared the effects of the Ab between TSOD mice and T.B-*Nidd5*/3 mice, which harbor an ALK7 nonsense mutation in the same genetic background (5, 16). Although administration of ALK7 mAb did not alter food intake or BW in either mouse strain during this 6-week treatment period, it potently reduced adiposity of TSOD mice to levels comparable to those in T.B-*Nidd5*/3 mice (Figure 1A). Fat pad weights and serum leptin concentrations were also markedly reduced in Ab-treated TSOD mice to levels equivalent to those found in T.B-*Nidd5*/3 mice (Figure 1, B and C). ATGL protein levels were upregulated in epiWAT (Figure 1D), and serum levels of nonesterified fatty acids (NEFAs) and glycerol normalized to epiWAT weight tended to increase in ALK7 mAb–treated TSOD mice (Figure 1E), suggesting increased lipolysis. However, the serum levels of NEFA and glycerol were not changed, possibly due to the resultant decrease in fat mass. Importantly, these effects of the Ab were not observed in ALK7-deficient T.B-*Nidd5*/3 mice (Figure 1, A–E), suggesting that the observed effects were due to ALK7 inhibition.

Consistently, the Ab specifically bound ALK7, but not ALK4 or ALK5, expressed in HEK293T cells (Supplemental Figure 1E), in accordance with the recent finding that this Ab blocks activin B and activin C signaling via ALK7 partly and almost completely, respectively, but does not affect activin A signaling via ALK4, in HEK293T cells expressing a luciferase reporter containing a Smad2/3 responsive element (17). These results indicate that the ALK7 mAb specifically and nearly completely blocks ALK7 function in vivo.

To investigate whether an extended dosing regimen would exert similar effects, we administered ALK7 mAb to TSOD mice at the same dose level and frequency for 15 weeks. Under these conditions, ALK7 mAb treatment reduced BW significantly and reduced adiposity by 50% compared with vehicle-treated TSOD mice (Figure 1F). Mice treated with ALK7 mAb had profound decreases (40%–60%) in fat pad weights (Figure 1G) and a proportional decrease in serum leptin levels (Figure 1H). Serum levels of NEFAs and glycerol normalized to epiWAT weight were increased, although the serum levels themselves did not increase but instead tended to decrease (Figure 1I).

**Figure 2:**
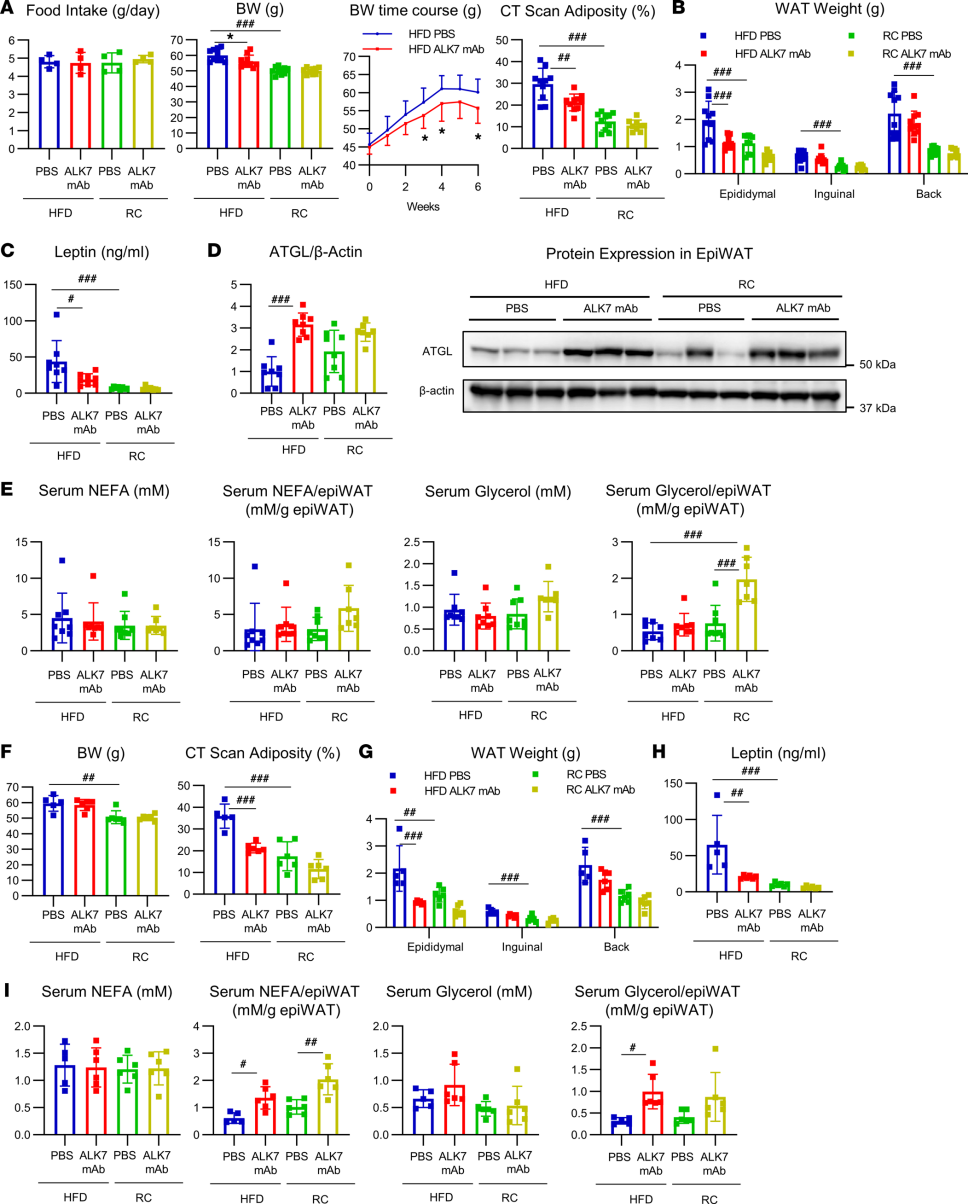
ALK7 mAb treatment reduces adiposity in outbred ddY mice fed an HFD but not in mice fed regular chow. Outbred ddY mice were fed either an HFD or regular chow (RC) from 4 weeks of age. After HFD-fed mice reached a weight of 45 g, on average at 7 weeks of age, mice started to receive s.c. injections of ALK7 mAb (10 mg/kg) or PBS for 6 weeks (**A**, *n* = 4 for food intake and *n* = 9 or 10 for others; **B**, *n* = 9 or 10; **C**–**E**, *n* = 7 or 8) or 18 weeks (**F**–**I**, *n* = 5 or 6). Phenotypic analyses were performed as described in Figure 1. (**D**) A representative ATGL and β-actin blot of 3 mice per group is shown. CT was analyzed at 5 and 14 weeks after the start of injections for the 6- and 18-week cohorts, respectively. Others were analyzed at the end of the experiments. ^#^*P* < 0.05, ^##^*P* < 0.01, ^###^*P* < 0.001 by 1-way ANOVA. *P < 0.05; *t* test.

To further investigate the potential of the ALK7 Ab treatment for adiposity, we next administered the Ab to a different mouse model of obesity: an outbred ddY strain fed an HFD, which displays marked postprandial hypertriglyceridemia in response to dietary fat (18). We started the HFD when the mice were 4 weeks of age, and the Ab treatment was started when they were 7 to approximately 9 weeks of age, when the BWs averaged 45 g. Again, the Ab injected only 1 time accumulated in epiWAT and upregulated the protein level of ATGL maximally on days 2 and 3 and did not increase serum LDH activity (Supplemental Figure 1, F and G). Although the Ab treatment for 6 weeks did not affect food intake, it resulted in modestly decreased BW and markedly decreased adiposity, fat pad weights, and serum leptin concentrations, specifically in mice fed an HFD, but not in those fed regular chow (RC) (Figure 2, A–C). It also upregulated the protein level of ATGL in epiWAT of mice fed an HFD (Figure 2D). The serum levels of NEFAs and glycerol normalized to epiWAT weight tended to increase, although their serum levels did not differ (Figure 2E). An extended treatment for 18 weeks also showed similar and more profound effects on the phenotypes described above, except BW (Figure 2, F–I).

### ALK7 mAb improves glucose tolerance and insulin sensitivity by increasing fat utilization and energy expenditure.

**Figure 3:**
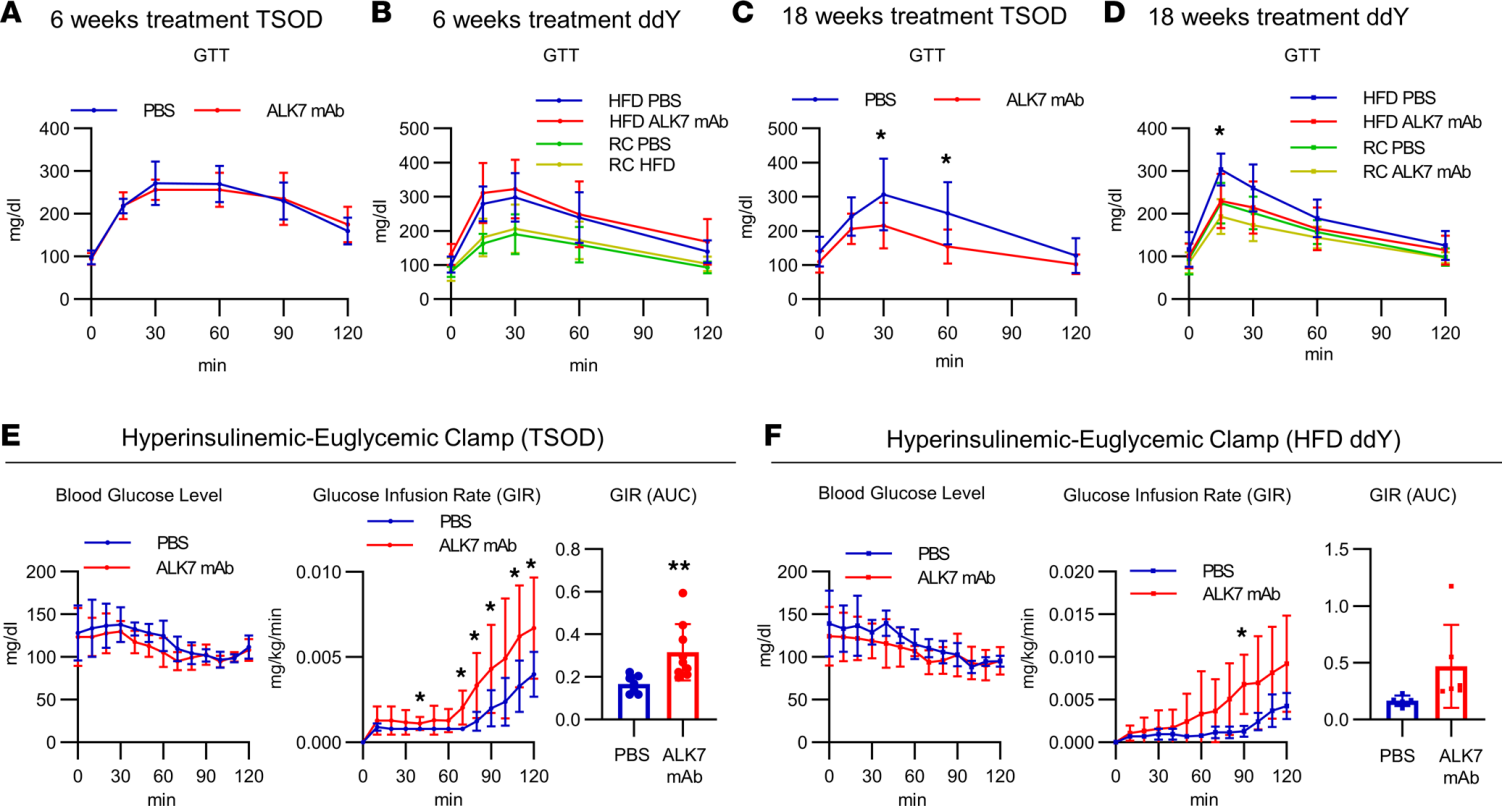
Long-term ALK7 mAb treatment improves glucose tolerance and insulin sensitivity in obese mice. (**A**–**D**) TSOD and HFD-fed ddY mice were treated with ALK7 mAb or PBS for 6 (**A**, *n* = 8; **B**, *n* = 7 or 8 per group) or 18 weeks (**C**, *n* = 9; **D**, *n* =5 or 6), as described in Figures 1 and 2. Blood glucose levels from the i.p. glucose tolerance test (GTT) after 14 hours of fasting were examined at 5 and 14 weeks after the start of injections for the 6- and 18-week cohorts, respectively, in TSOD (**A** and **C**) and ddY mice fed an HFD (**B** and **D**). Shown are the glucose levels, glucose infusion rates (GIRs), and total amounts of glucose infused (AUC) during a hyperinsulinemic-euglycemic clamp test in TSOD mice (**E**) and ddY mice fed an HFD (**F**) after 17 to 18 weeks of treatment. *P < 0.05, **P < 0.01 by *t* test. RC, regular chow.

Reflecting the reduced adiposity, ALK7-deficient T.B-*Nidd5*/3 mice display improved glucose tolerance and insulin sensitivity compared with their WT counterparts, TSOD mice (5). We investigated whether the Ab treatment could induce similar effects. Although the shorter 6-week treatment did not change glucose tolerance in either TSOD or ddY mice fed an HFD (Figure 3, A and B), the longer 14- to 18-week treatment markedly enhanced it in both strains of mice (Figure 3, C and D). Hyperinsulinemic-euglycemic clamp tests revealed increased insulin sensitivity in mice treated for the longer periods (Figure 3, E and F). It was suggested that ALK7 dysfunction in β cells primarily causes late-onset insulin resistance in ALK7-KO C57BL/6 mice (19). However, ALK7 was not expressed significantly in the pancreatic islets or whole pancreas of the C57BL/6N, ddY, and TSOD strains (Supplemental Figure 2, A and B). Furthermore, neither basal nor glucose-stimulated insulin secretion from isolated islets of TSOD mice was altered by in vivo ALK7 mAb treatment (Supplemental Figure 2C). These findings are consistent with previous findings that ALK7 expression is barely detectable in islets of TSOD mice and that islets from ALK7-deficient T.B-*Nidd5*/3 mice show no changes in insulin secretory ability compared with those from ALK7-intact TSOD mice (5).

**Figure 4:**
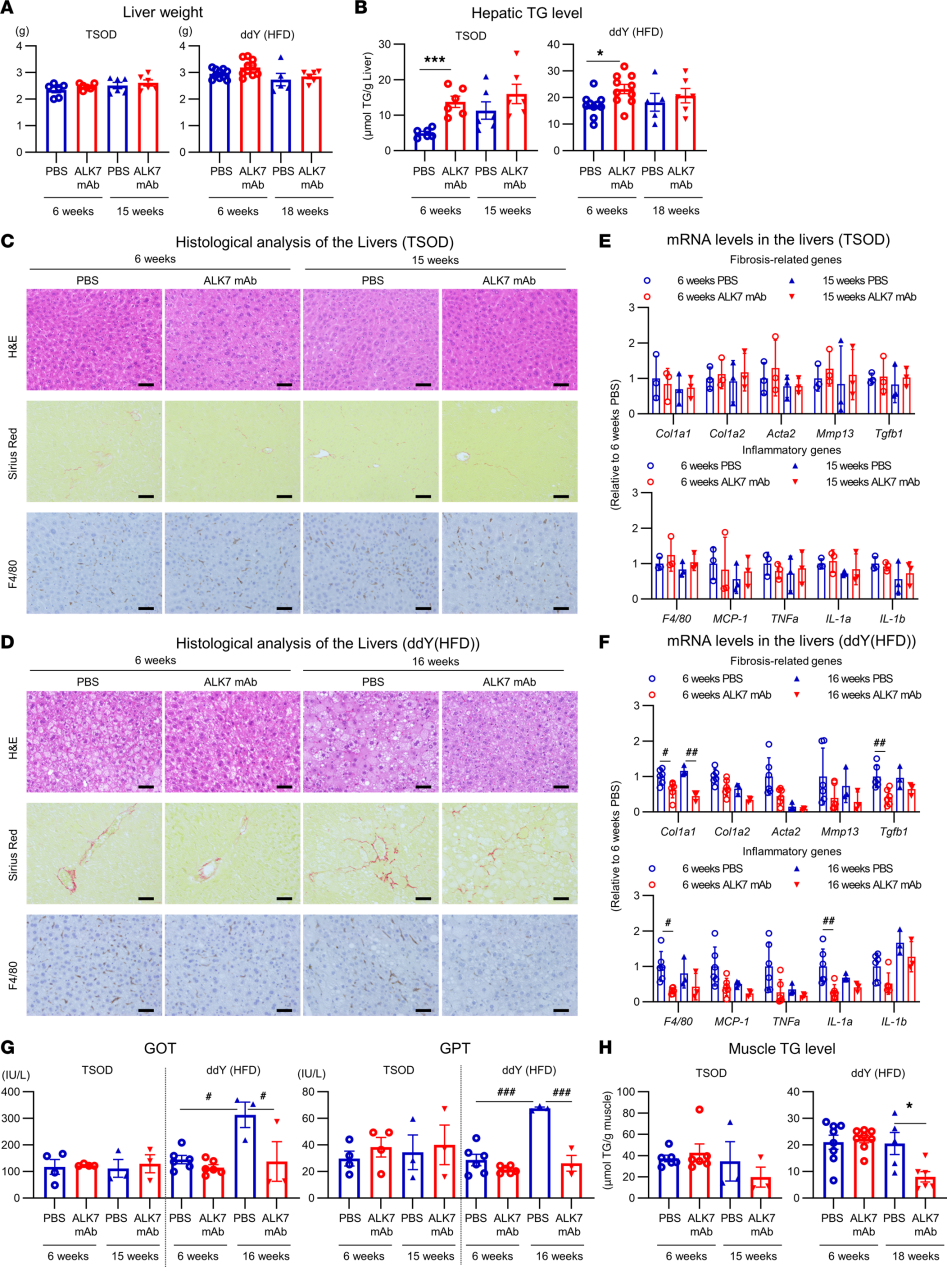
Long-term ALK7 mAb treatment does not increase ectopic fat accumulation in liver or muscle. TSOD or HFD-fed ddY mice were treated with ALK7 mAb or PBS for the indicated time, as described in Figures 1 and 2. (**A** and **B**) Weight (**A**), and TG content (**B**) of liver (*n* = 6 to approximately 10 per group). (**C** and **D**) H&E, Sirius Red, and IHC DAB staining against F4/80 (positive staining: brown) of liver. Shown are pictures representative of TSOD (**C**; *n* = 4 in 6-week cohort and *n* = 3 in 15-week cohort) and ddY mouse samples (**D**; *n* = 2 in 6-week cohort and *n* = 3 in 16-week cohort). Scale bars: 50 μm. (**E**–**G**) mRNA levels of fibrosis- and inflammation-related genes in liver (**E** and **F**) and serum GOT and GPT levels (**G**) of TSOD (*n* = 3–4 per group) and ddY mice (*n* = 6 in 6-week cohort and *n* = 3 in 16-week cohort). (**H**) TG content of type IIb–dominant white muscle in lower limbs; gastrocnemius muscle from TSOD mice (*n* = 3–6) and gastrocnemius muscle from 6-week cohort of ddY mice and tibialis anterior muscle from 18-week cohort of ddY mice (*n* = 5–8). *P < 0.05, ***P < 0.001 by *t* test. ^#^*P* < 0.05, ^##^*P* < 0.01, ^###^*P* < 0.001, 1-way ANOVA.

To explore the basis for these time-dependent changes in glucose tolerance, we investigated the effects of the Ab in several peripheral tissues that have major influences on whole-body metabolism. Because the reducing effects of the Ab on BW are relatively small compared with the effects on adiposity, it is possible that lipids released from adipocytes by increased lipolysis may be redistributed to other peripheral tissues. In fact, ALK7-KO mice have been shown to display marked liver steatosis when fed an HFD (4) and at older ages, even under a regular chow diet (19). Indeed, the neutralizing Ab treatment for 6 weeks markedly increased hepatic triglyceride (TG) levels in both TSOD and ddY mice fed an HFD, although liver weight was not altered (Figure 4, A and B). However, the longer 15- to 18-week treatment largely erased these differences, which may account for the improved glucose tolerance and insulin sensitivity (Figure 3, C–F). Furthermore, histological examination of liver revealed no signs of steatosis, fibrosis, or inflammation compared with control mice after either short- or long-period treatment (Figure 4, C and D).

Consistent with these findings, the Ab treatment did not elevate expression of fibrosis-related or inflammatory genes in liver or serum levels of glutamic oxaloacetic transaminase (GOT) or glutamic pyruvic transaminase (GPT) (Figure 4, E–G). In ddY mice, some levels of these markers were even decreased by the Ab treatment. Moreover, the longer treatment was associated with decreased TG levels in muscle, another large organ that ectopically accumulates lipids (Figure 4H). These findings suggest that lipids released from adipocytes by increased lipolysis transiently accumulate in other peripheral tissues, but are eventually consumed.

**Figure 5:**
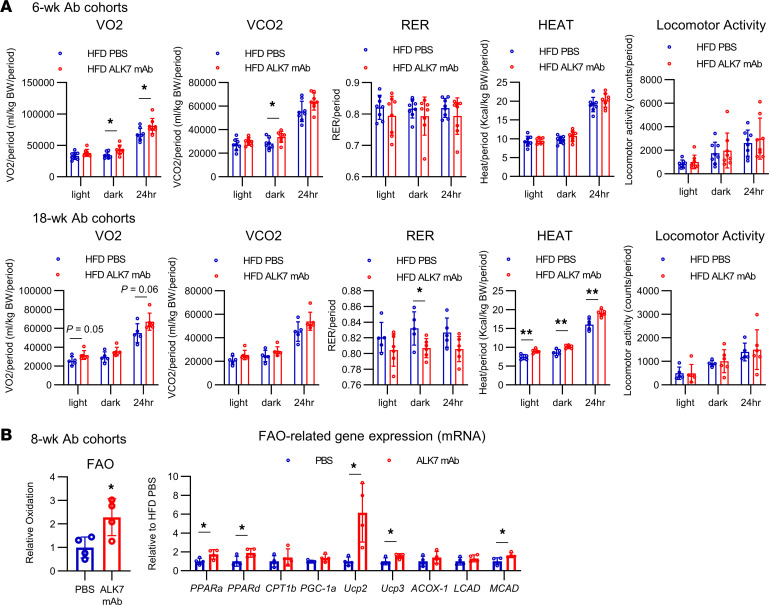
ALK7 mAb treatment increases energy expenditure, fat use, and heat production. (**A**) HFD-fed ddY mice were treated with ALK7 mAb or PBS, as described in Figure 2. Oxygen consumption (VO_2_), CO_2_ production (VCO_2_), respiratory exchange rate (RER), heat production, and locomotion activity in a metabolic cage during light, dark, and total 24-hour periods were measured after 5 weeks (see first 5 charts; *n* = 8) and 15 weeks (see second group of 5 charts; *n* = 5 or 6) of treatment. (**B**) FAO and mRNA levels of FAO-related genes in type I–dominant soleus muscle isolated from HFD-fed ddY mice treated with ALK7 mAb or PBS for 8 weeks (*n* = 4 each). *P < 0.05, **P < 0.01, *t* test.

We then investigated the Ab effects on fat utilization at the whole-body level by putting ddY mice fed an HFD in metabolic cages. As observed in ALK7-deficient T.B-*Nidd5*/3 mice (5), the Ab treatment for both 6 and 18 weeks increased oxygen consumption and, thus, energy expenditure (Figure 5A). Furthermore, respiratory exchange ratios tended to decrease, consistent with the findings that fatty acid oxidation (FAO) and levels of several molecules involved in this process (20) were increased in muscle in Ab-treated mice (Figure 5B). These findings suggest that NEFAs released from adipocytes by increased lipolysis is ultimately broken down by FAO and efficiently cleared from the serum.

We also noticed increased heat production after the longer term treatment (Figure 5A). ALK7 is expressed at relatively high levels in brown adipose tissue (BAT) (5), which is involved in nonshivering thermogenesis. Mice lacking ALK7 in brown adipocytes expressing Cre recombinase under the regulatory sequence of the Ucp1 gene had reduced BAT mass after 14 hours of fasting, but not under conditions of ad libitum chow (21). The Ab treatment did not significantly change BAT weights or UCP-1 expression under the nonfasting condition (Supplemental Figure 3A). Instead, UCP-1 and β_3_-adrenoreceptor were upregulated in Ab-treated WAT (Supplemental Figure 3B), which could contribute to the increases in heat production and energy expenditure in Ab-treated mice. The β_3_-adrenoreceptor has been reported to be upregulated in WAT of ALK7-KO mice (6).

### ALK7 mAb downregulates GDF3 in ATMs by decreasing production of S100A8/A9 in adipocytes and IL-1β in ATMs.

**Figure 6:**
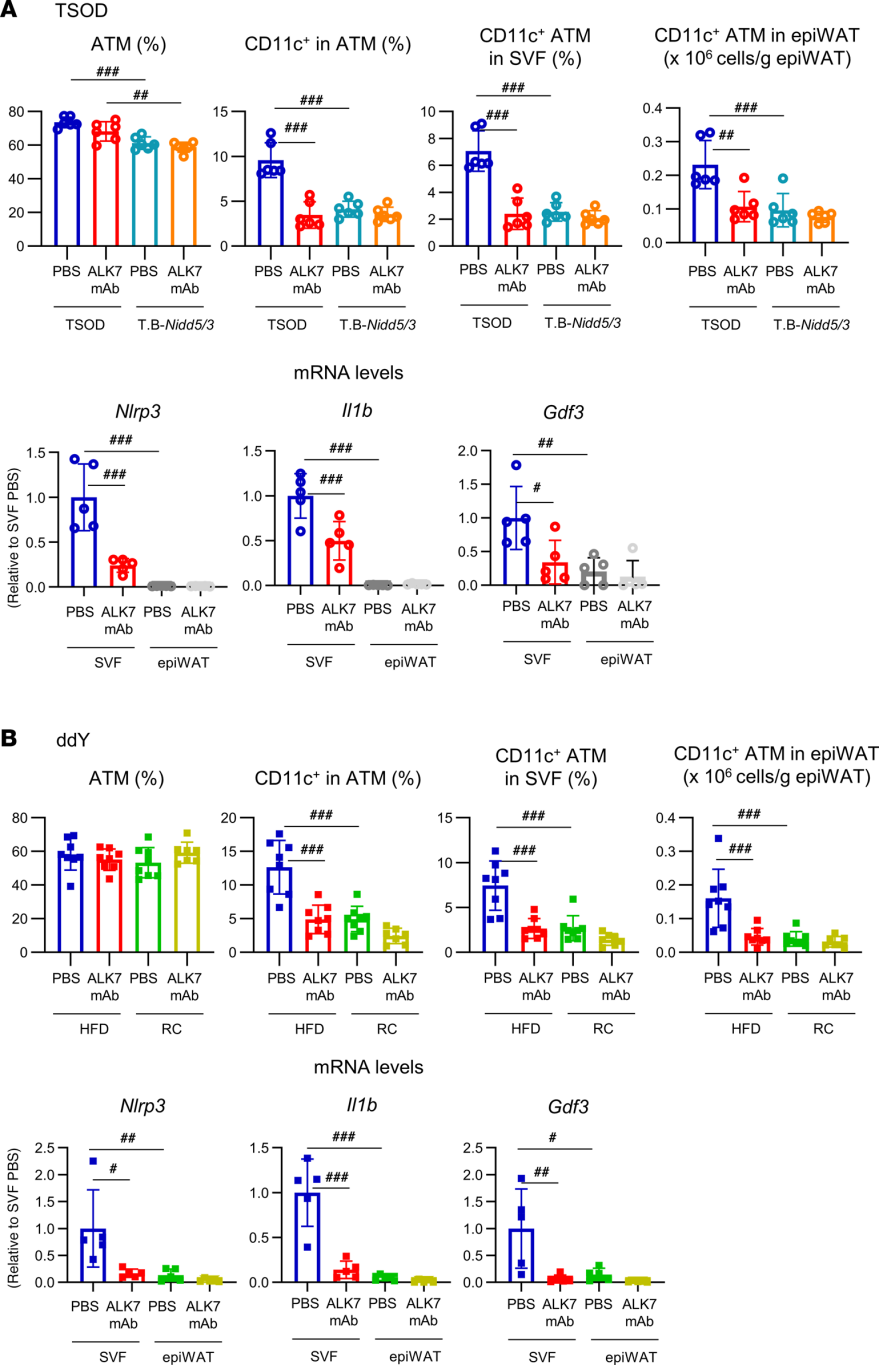
ALK7 mAb treatment reduces GDF3 expression in ATMs. (**A** and **B**) TSOD or T.B-*Nidd5*/3 mice (**A**) and ddY mice fed either an HFD or regular chow (RC) (**B**) were treated with ALK7 mAb or PBS for 6 weeks, as described in Figures 1 and 2. Then, the SVF was isolated from epiWAT, and the cell differentials were determined by FACS (see first 4 charts). Shown are percentages of ATMs (CD11b^+^F4/80^+^) in SVF cells, CD11c^+^ cells in ATMs, and CD11c^+^ ATMs in SVF cells, and numbers of CD11c^+^ ATMs normalized by fat weight from TSOD or T.B-*Nidd5*/3 mice (**A**, *n* = 5 or 6) and from HFD- or RC-fed ddY mice (**B**, *n* = 7 or 8). mRNA levels of NLRP3 (*Nlrp3*), IL-1β (*Il1b*), and GDF3 (*Gdf3*) were determined by real-time reverse transcription PCR (RT-PCR) in epiWAT and its SVF (see the last 3 charts in **A** and **B**; *n* = 5or 6 per group). ^#^*P* < 0.05, ^##^*P* < 0.01, ^###^*P* < 0.001 by 1-way ANOVA.

We previously determined that CD11c^+^ ATMs express the ALK7 ligand GDF3 and that the number of GDF3^+^ cells is reduced in WAT of ALK7-deficient T.B-*Nidd5*/3 mice (7), despite negligible expression of ALK7 in ATMs (5). Analysis of the stromal vascular fraction (SVF) of epiWAT revealed that the percentage of CD11c^+^ ATMs among total ATMs (CD11b^+^F4/80^+^) or SVF cells and their number were markedly reduced in ALK7 mAb–treated TSOD mice and were similar to the levels found in T.B-*Nidd5*/3 mice (Figure 6A). The percentage and the number of CD11c^+^ ATMs were also decreased in ALK7 mAb–treated ddY mice fed an HFD and were similar to the levels found in those mice fed regular chow (Figure 6B). These data support the concept that decreased production of GDF3 in adipose tissue further contributes to the observed reduction in adipose mass in ALK7 mAb–treated mice. The reduction in GDF3-producing cells in ALK7 mAb–treated mice was accompanied by decreased expression of inflammasome-related genes such as *Nlrp3* and *Il1b* in the SVF (Figure 6, A and B), in accordance with a previous finding that deletion of *Nlrp3* in mouse ATMs downregulates GDF3 (9).

**Figure 7:**
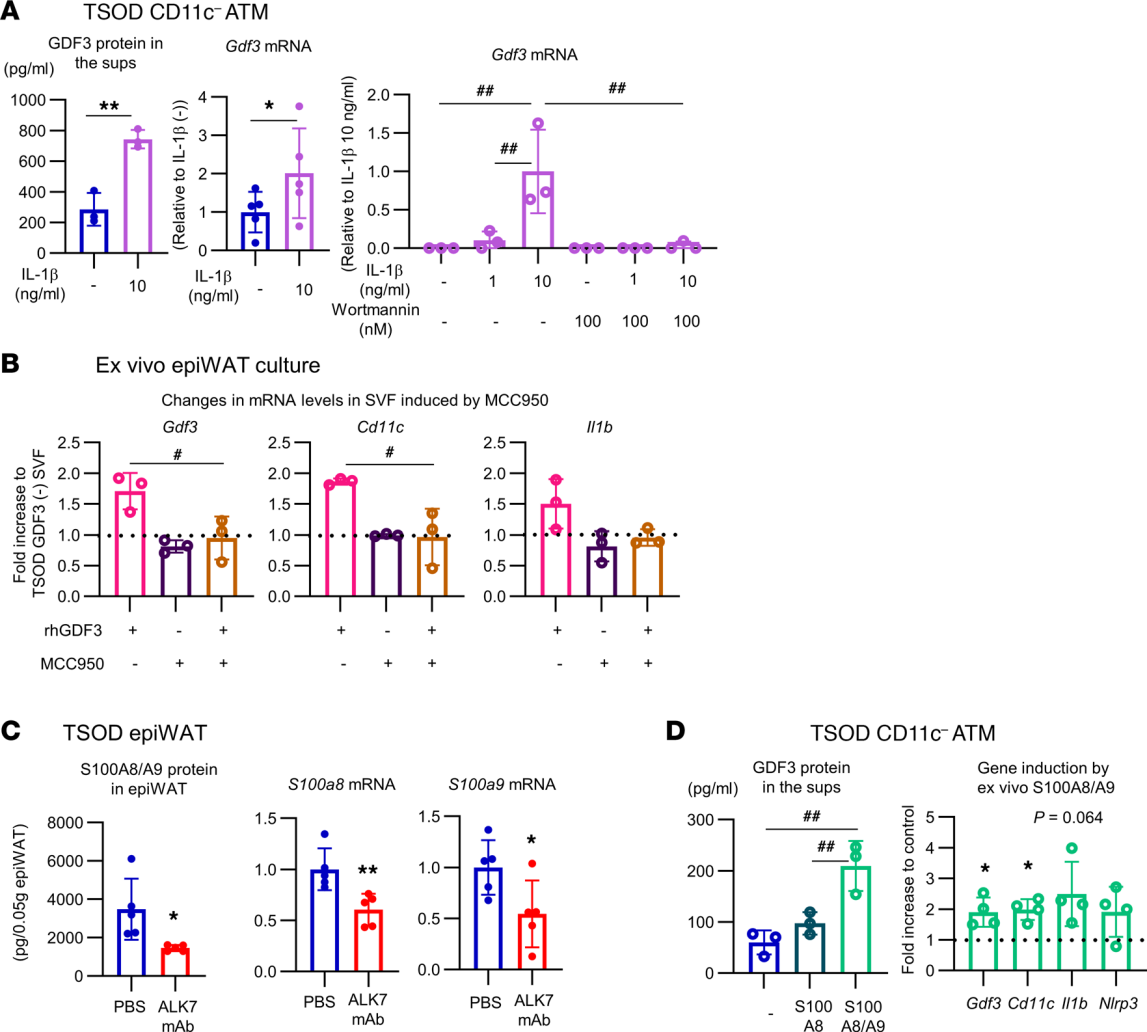
IL-1β from ATMs and S100A8/A9 from adipocytes mediate a positive feedback loop in the GDF3/ALK7 axis. (**A**) CD11c^–^ ATMs purified by FACS from epiWAT SVF of 7- to 9-week-old TSOD mice were plated in a 24-well dish and incubated with or without the recombinant mature form of mouse IL-1β (10 ng/mL) in the absence of FBS. The PI3K inhibitor wortmannin (100 nM) was added 10 minutes before the addition of IL-1β (see third chart). After a 24-hour culture, GDF3 protein concentrations in the supernatants (sups) (first chart, *n* = 3) and GDF3 mRNA levels in the cell pellets (second chart, *n* = 5; third chart, *n* = 3) were measured by real-time ELISA and RT-PCR, respectively. (**B**) EpiWAT (0.5 g) isolated from 7-week-old TSOD mice were incubated with or without rrhGDF3 (400 ng/mL) in the presence of 20% FBS. The NLRP3 inhibitor, MCC950 (10 μM), was also added into some wells 30 minutes before the addition of GDF3. After a 24-hour culture, the mRNA levels in the SVF isolated from approximately 0.35 g of epiWAT were determined (*n* = 3). (**C**) TSOD mice were treated with ALK7 mAb (10 mg/kg) or PBS for 6 weeks, as described in Figure 1. The expression levels of S100A8/A9 heterodimer protein and those of S100A8 and S100A9 mRNA in epiWAT were determined by ELISA and real-time RT-PCR, respectively (*n* = 5). (**D**) CD11c^–^ ATMs (1.0 × 10^6^ cells/24-well dish) isolated from epiWAT SVF of 7- to 8-week-old TSOD mice were cultured with or without S100A8 or S100A8/A9 at 10 μg/mL. After a 24-hour culture, the GDF3 protein and the mRNA levels were measured (*n* = 3), as described in **A** and **B**. *P < 0.05, **P < 0.01 by *t* test. ^#^*P* < 0.05, ^##^*P* < 0.01 by 1-way ANOVA.

The above findings suggest the presence of reciprocal positive-feedback signals between ATMs and adipocytes, in which unidentified ALK7-dependent signals from adipocytes stimulate inflammasome-driven GDF3 production in ATMs. IL-1β is one of the major targets of caspase-1 activated by the NLRP3 inflammasome. To explore the possibility that IL-1β secreted from ATMs cell-autonomously upregulates GDF3, we added a recombinant mature form of IL-1β to CD11c^−^ ATMs from TSOD mice ex vivo. IL-1β dose-dependently increased GDF3 production in ATMs, which was inhibited by preincubation with the PI3K inhibitor wortmannin (Figure 7A). Furthermore, recombinant GDF3 protein added to epiWAT ex vivo increased the expression of GDF3, CD11c, and IL-1β, which was inhibited by prior incubation with the NLRP3 inhibitor MCC950 (22) (Figure 7B). These findings suggest the involvement of NLRP3 and downstream IL-1β in GDF3-induced GDF3 expression in WAT.

To explore the nature of the ALK7-dependent signals from adipocytes that induce GDF3 expression in ATMs, we focused on adipose S100A8/A9 proteins, which have been suggested as possible initiating ligands that stimulate ATMs to activate the NLRP3/inflammasome/IL-1β pathway (23). We found that both S100A8 and S100A9 mRNAs and S100A8/A9 protein are downregulated in epiWAT of ALK7 mAb–treated TSOD mice (Figure 7C). Their expression occurred mainly in the adipocytes, whereas that in SVF was not affected by in vivo ALK7 mAb treatment (Supplemental Figure 4A), consistent with the lack of ALK7 in ATMs (Supplemental Figure 4B). Furthermore, incubation of CD11c^–^ ATMs with S100A8/A9 promoted production of GDF3 with upregulation of CD11c, IL-1β, and NLRP3 (Figure 7D).

**Figure 8:**
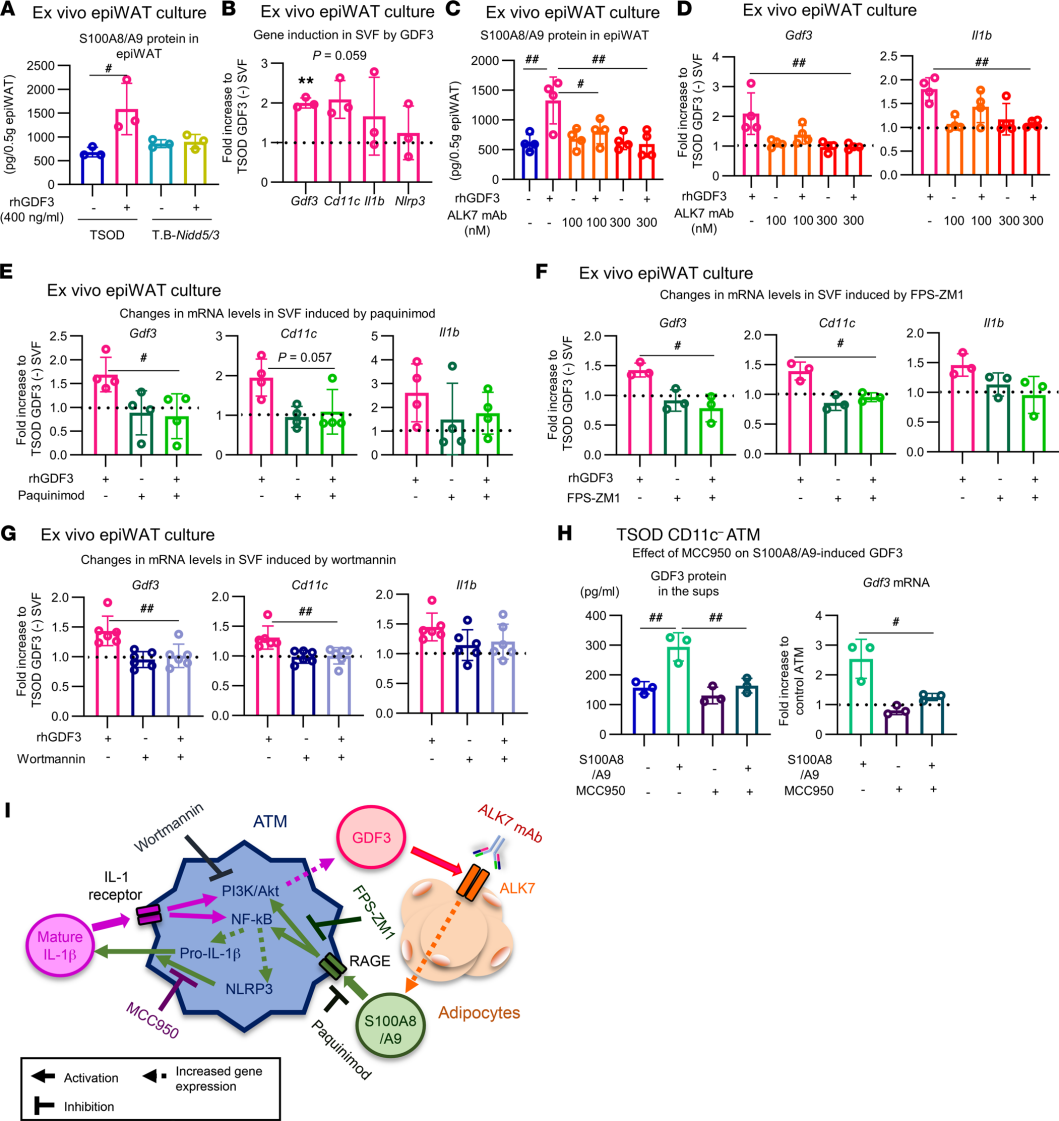
Signaling pathways of S100A8/A9-induced GDF3 upregulation in ATMs. (**A**–**G**) EpiWAT (0.5 g) isolated from 7-week-old TSOD or T.B-*Nidd5*/3 mice was incubated with GDF3, as described in Figure 7B. (**C**–**G**) ALK7 mAb (**C** and **D**; 30 minutes), the S100A8/A9 inhibitor, paquinimod (**E**; 900 μg/mL, 10 minutes), the RAGE antagonist, FPS-ZM1 (**F**; 30 μg/mL, 2 hours), or wortmannin (**G**; 100 nM, 10 minutes) was added at the indicated concentrations prior to the addition of GDF3. After a 24-hour culture, RNA and protein were extracted from 0.05 g and 0.1 g of epiWAT, respectively. SVF was isolated from the remaining approximately 0.35 g of epiWAT. The S100A8/A9 protein levels were measured as described in Figure 7C (**A**, *n* = 3; **C**, *n* = 4). The mRNA levels in SVF were determined (**B**, *n* = 3; **D**, *n* = 4; **E**, *n* = 4; **F**, *n* = 3; **G**, *n* = 6). (**H**) CD11c^–^ ATMs isolated from epiWAT SVF of 7- to 10-week-old TSOD mice were cultured with or without MCC950 (10 μM) for 30 minutes followed by S100A8/A9 (10 μg/mL) for 24 hour. The GDF3 protein and its mRNA levels were measured (*n* = 3), as described in Figure 7A. (**I**) Schematic summary. GDF3 increases production of S100A8/A9 by adipocytes through its receptor ALK7. S100A8/A9 increases production of pro–IL-1β in ATMs. Pro–IL-1β can be cleaved to form bioactive mature IL-1β by NLRP3 inflammasome. S100A8/A9 may also directly enhance GDF3 production by ATMs via activation of PI3K. Secreted mature IL-1β increases GDF3 production by ATMs in autocrine and/or paracrine manners via a PI3K-dependent pathway. As such, GDF3 released from ATMs and ALK7 signals in adipocytes forms a positive feedback loop to drive fat accumulation. ^#^*P* < 0.05, ^##^*P* < 0.01 by 1-way ANOVA. **P < 0.01 by *t* test.

Conversely, ex vivo treatment with recombinant GDF3 protein increased production of S100A8/A9 protein from epiWAT of TSOD mice, but not from ALK7-deficient T.B-*Nidd5*/3 mice (Figure 8A). This induction of S100A8/A9 by GDF3 did not occur in SVF (Supplemental Figure 4C). Furthermore, clodronate treatment to deplete macrophages significantly decreased the expression of F4/80, as described (7), but did not affect the induction of S100A8/A9 heterodimer protein by GDF3 in epiWAT (Supplemental Figure 4D). These findings confirm that the upregulation of S100A8/A9 by GDF3 occurs via ALK7 in adipocytes. Ex vivo GDF3 treatment upregulated CD11c, IL-1β, and NLRP3 as well as GDF3 itself in epiWAT of TSOD mice, but not in that of T.B-*Nidd5*/3 mice (Figure 8B and Supplemental Figure 4E). Furthermore, preincubation of ALK7 mAb inhibited GDF3-induced upregulation of S100A8/A9 (Figure 8C), as well as that of GDF3, CD11c, IL-1β, and NLRP3 (Figure 8D and Supplemental Figure 4F), and blocked GDF3-induced Smad3 phosphorylation (Supplemental Figure 4G). Similar GDF3-induced gene induction ex vivo was found in epiWAT of ddY mice (Supplemental Figure 4H).

S100A8/A9 can induce inflammatory cytokines by engaging pattern recognition receptors, including TLR4 (24) and the receptor for advanced glycation end products (RAGE) (25). Administration of paquinimod, which inhibits S100A9 interactions with TLR4 and RAGE (26), to epiWAT of TSOD mice ex vivo markedly downregulated GDF3-induced upregulation of GDF3, CD11c, and IL-1β (Figure 8E). Furthermore, treatment of epiWAT with the RAGE antagonist FPS-ZM1 phenocopied that with paquinimod (Figure 8F), whereas treatment of epiWAT with LPS acting on TLR4 did not upregulate GDF3 expression (Supplemental Figure 4I). This finding suggests that S100A8/A9 functions via RAGE in this context, although we cannot eliminate the possibility that it functions via the combination of RAGE and TLR4. Such GDF3-induced gene upregulation reverted to the baseline by treatment of epiWAT with wortmannin (Figure 8G). Furthermore, S100A8/A9-induced GDF3 production in CD11c^–^ ATMs was inhibited by MCC950 pretreatment (Figure 8H), suggesting the involvement of NLRP3 and IL-1β in this process. Taken together, our data suggest that a mechanism of reciprocal positive-feedback signals between ATMs and adipocytes may function as follows (Figure 8I): GDF3 released from ATMs binds ALK7 and induces production of S100A8/A9 protein from adipocytes, which, in turn, activates NLRP3 and promotes release of mature IL-1β from ATMs that cell-autonomously increases expression and release of GDF3 in ATMs and further activates ALK7-mediated pathways in adipocytes to promote adiposity.

## Discussion

In the present study, we showed that a neutralizing ALK7 Ab decreases fat mass profoundly (40%–60%) in genetically determined obese and diabetic TSOD mice, and in outbred ddY mice fed an HFD. The Ab seems to completely block ALK7 action in vivo, because the residual adipose tissue mass after treatment in TSOD mice was equal to that in TSOD mice containing the ALK7 nonsense mutation. Moreover, the ALK7 Ab treatment did not result in reduced fat mass in TSOD mice harboring the ALK7 nonsense mutation, suggesting that the Ab’s effect is mediated by specific inhibition of ALK7 signaling. These findings provide compelling evidence that the ALK7 signaling pathway plays a major role in the regulation of lipid metabolism and fat accumulation in the whole body and that anti-ALK7 treatment holds promise as a robust and effective intervention for adiposity with diverse causes.

Consistent with the phenotypes of genetically ALK7-deficient mice (5, 7), the blockade of ALK7 signals by the Ab led to upregulation of lipolysis, which decreased fat mass in adipocytes. This may be a useful strategy for combating obesity, because stored fat must first be degraded by lipolysis to reduce adiposity. However, increased lipolysis in a basal state has conventionally been thought to increase the levels of circulating NEFA and induce insulin resistance (27, 28). The Ab treatment discussed in the present report, however, induced no changes in the serum levels of NEFA and glycerol, despite the increased lipolysis. This likely results from reduced fat mass induced by the treatment, because the rate of lipid removal through lipolysis is known to be mediated proportionally by both the total fat mass and the activities of lipases (8). There are discrepant findings in previous reports about the effects of expressional changes in adipose lipases in mice. Partial or incomplete inhibition of adipose lipases has been shown to improve insulin sensitivity in HFD-fed mice (29, 30). By contrast, mice overexpressing ATGL in WAT moderate diet-induced obesity by promoting FAO and re-esterification within adipocytes, which result in smaller adipocyte sizes and higher insulin sensitivity without increases in serum NEFA levels or ectopic TG storage (31). In humans, the rate of lipid removal is positively correlated with the capacity of adipocytes to break down TG by lipolysis and is inversely related to insulin resistance (32). These discrepant findings at least indicate that the relationship between increased lipolysis and metabolic effects is not straightforward and may be context dependent. It should be remembered, however, that ALK7 deficiency does not directly upregulate adipose lipases but does upregulate C/EBPα and PPARγ, which activate both TG synthesis and breakdown (5). Although the rate of lipolysis may exceed that of TG synthesis when fat mass and adipocyte sizes remain large in the initial phase of Ab treatment, fat mass and lipid release from adipocytes will decline as treatment continues (8). In fact, we showed that anti-ALK7 treatment improves glucose tolerance and insulin sensitivity after 14 to 18 weeks of treatment, but not after a 6-week treatment.

A serious concern with increased lipolysis is ectopic TG storage in muscle and liver due to increases in lipids released from adipocytes (33, 34). In fact, ALK7-deficient C57BL/6 mice fed an HFD exhibit liver steatosis and insulin resistance (19). We showed that, although anti-ALK7 treatment for 6 weeks increased hepatic TG content, the same treatment for 16–18 weeks normalized hepatic TG content and even decreased muscle TG content. The discrepancy could be partly explained by differences in genetic background of the mice being studied, because C57BL/6 mice are known to be susceptible to HFD-induced obesity, insulin resistance, and nonalcoholic fatty liver disease (35). However, the time-dependent effects of neutralizing ALK7 Ab in the same strain shown here indicate that the lipids released from adipocytes and that accumulate in other tissues may eventually be consumed. In fact, anti-ALK7 treatment induces preferential NEFA oxidation in peripheral tissues, increased energy expenditure at the whole-body level, and eventual reduction in fat and ectopic TG mass. Although the mechanism by which ALK7 blockade increases FAO is unknown, some lipid components released from adipocytes by increased lipolysis may increase the activities of PPARα and/or PPARδ, as well as their expression levels, as observed in the present study, which are known to increase FAO. Consistent with this view, it has recently been reported that ATGL-dependent WAT lipolysis controls PPARα activity in liver (36). Given the reported increases in CA availability by GDF3 deficiency (9) and in CA sensitivity by ALK7 deficiency (6), anti-ALK7 treatment may also increase CA-induced lipolysis, which is generally thought to be beneficial for whole-body metabolism (37, 38).

Another concern with increased lipolysis is lipodystrophy and associated insulin resistance (39). However, ALK7 mAb treatment did not induce any lipodystrophic changes in mice, including ddY mice fed regular chow. Consistently, congenitally ALK7-deficient T.B-*Nidd5*/3 mice had smaller adipocytes without changes in the number of either WAT or BAT cells compared with ALK7-intact TSOD mice (16). Furthermore, BALB/c strains harboring the nonsense mutation had no severe lipodystrophy or insulin resistance (5). This may be because the ALK7 signal is activated primarily under nutrient-excess conditions, which is suggested by the finding that ALK7-KO mice fed regular chow exhibited no changes in fat weight in contrast to those fed an HFD (4). This hypothesis is further supported by the finding that the ALK ligand GDF3 is upregulated by insulin, which is secreted in response to nutrient load (7). Moreover, considering that blocking of the ALK7 signals upregulates the adipose master regulators, C/EBPα and PPARγ (5), it should not induce unlimited lipolysis resulting in lipodystrophy. It has recently been shown that mice lacking ALK7 in BAT exhibit fasting-induced hypothermia due to exaggerated catabolic activity in brown adipocytes when exposed to 5°C for 4 hours (21). However, at least under a normal thermoneutral breeding environment, we found no change in BAT levels in ALK7 Ab–treated mice. We instead found significant upregulation of UCP-1 and β_3_-adrenoreceptor genes in white adipocytes, suggesting browning of the WAT (40, 41).

Obesity is associated with mild chronic inflammation elicited in part by increased pro-inflammatory cytokines and accumulation of ATMs (42). However, the specific sequence of cellular interactions underlying this process is unknown. The present study showed that an ALK7-neutralizing Ab treatment decreased the number of GDF3^+^ ATMs specifically in ALK7-intact TSOD mice, consistent with the previous finding that number of GDF3^+^ cells is reduced in adipose tissue of ALK7-deficient TSOD mice (7). Because ALK7 is barely detectable in ATMs, ALK7-dependent signals from adipocytes appear to stimulate GDF3 production in ATMs. We showed involvement of the S100A8/A9/NLRP3/IL-1β axis in this reciprocal activation pathway from the receptor, ALK7, to the ligand, GDF3. First, ALK7 signals upregulate production and release of S100A8/A9 in adipocytes, possibly due to inhibition of PPARγ (5), because the PPARγ agonist has been shown to downregulate S100A8 (43). This S100A8/A9 then upregulates NLRP3 and IL-1β in ATMs via binding to RAGE. RAGE activates both PI3K and NF-κB pathways (44), whereas TLR4 mainly activates the NF-κB pathway (45). This difference in downstream pathways between RAGE and TLR4 may explain why S100A8/A9-RAGE signaling increases GDF3 expression in ATM but LPS-TLR4 signaling fails to do so.

NLRP3 activation converts IL-1β from the proform to the mature form by caspase-1 activation, and the released IL-1β upregulates GDF3 in ATMs via the PI3K pathway, in a manner similar to insulin (7). IL-1β further activates NLRP3 cell-autonomously (10). Interestingly, GDF3 is downregulated after genetic ablation of NLRP3 in ATMs (9). We showed that GDF3 is downregulated by treatment with either S100A8/A9 inhibitor or RAGE inhibitor, as well as by treatment with NLRP3 inhibitor. Thus, an ALK7-neutralizing Ab would be expected to induce simultaneous blockade of the receptor signal and the ligand production, which should increase the potency of this treatment and might prevent weight rebound even after discontinuation of treatment, because of reduced production of the upstream ligand.

The present findings demonstrate that anti-ALK7 treatment effectively targets fat accumulation in obesity across diverse physiologic contexts while sparing lean mass, although only male mice were phenotypically characterized and thus the relevance to female mice is unknown. Reflecting that the blockade of ALK7 signals does not directly decrease food intake or increase energy use but induces preferential use of lipids among nutrients, the reducing effects of the Ab on BW are relatively small compared with those on adiposity. However, except in cases with extreme obesity, the most important purpose of obesity treatment is not the BW reduction per se but the prevention of obesity-associated diseases. Three human *ACVR1C* variants harboring missense mutations in the receptor intracellular domain were recently associated with a reduced waist to hip ratio and resistance to diabetes (12), which indicates that the ALK7 signaling pathway serves a metabolic role that is conserved between rodents and humans. The anti-ALK7 treatment reported here offers promise for treating obesity and associated diabetes in humans. However, clinical studies testing ALK7-neutralizing Abs should incorporate markers to monitor for potentially undesirable side effects of ALK7 inhibition, including liver steatosis (19), female reproductive changes (15), and cardiac abnormalities (46, 47), that have been noted in ALK7-deficient C57BL/6 mice. It is currently unknown whether these adverse effects are specific to C57BL/6 mice. More research, including clinical studies of the therapeutic potential of the human ALK7 mAb, should be pursued to address obesity.

## Methods

### Animal procedures.

The TSOD mouse strain was originally established from an outbred ddY strain as an inbred strain with obesity and urinary glucose (13, 14). The congenic mouse strain, T.B-*Nidd5*/3, harboring a mutation inactivating the kinase activity of ALK7, was developed and characterized previously (5, 16). The ddY and C57BL/6N mice were purchased from Japan SLC and CLEA Japan, respectively. Mice had ad libitum access to standard laboratory chow (CE-2; CLEA Japan). To prepare diet-induced obese mice, an HFD (caloric percentages: 55% fat, 28% carbohydrate, 17% protein; Oriental Yeast Co., Ltd.) was given to ddY mice from 4 weeks of age. Purified anti-ALK7 human mAb containing a murine IgG2a-Fc domain was generated and provided by Acceleron Pharma. Control mouse IgG2a isotype Ab was purchased from Selleckchem (catalog A2117). Mice were dosed twice per week by s.c. injection with ALK7 mAb or vehicle (PBS) for the indicated duration. Injection started for TSOD and T.B-*Nidd5*/3 mice at 5 weeks of age, and for ddY mice at 7 to approximately 9 weeks of age, when the average BW of the HFD-fed mice reached 45 g. For macrophage depletion, liposomes containing 110 mg/kg BW clodronate or liposomes alone (ClodronateLiposomes.org) were injected i.p. twice per week for 3 weeks, as described previously (7).

Only male mice were phenotypically characterized in the present study. The whole-body composition was analyzed by CT (Latheta LCT-200; Hitachi). A glucose tolerance test was performed after an i.p. injection of glucose 1 g/kg BW. A hyperinsulinemic-euglycemic clamp test was performed after overnight fasting, as described previously (48). Insulin (10 mU/kg/min) was continuously infused into mice along with compensatory glucose infusion to maintain a blood glucose level of approximately 100 mg/dL.

For the insulin secretion assay, 10 fresh islets, isolated as described previously (5, 49), were first incubated at 37°C for 30 minutes in Krebs-Ringer HEPES buffer containing 0.1% BSA and 2.8 mM glucose, followed by another 30 minutes in the same buffer containing 16.7 mM glucose. The insulin concentrations secreted in the extracellular buffer, or the levels remaining in cells, were measured using an AlphaLISA insulin kit with an EnVision 2101 multilabel reader (PerkinElmer). Oxygen consumption, CO_2_ production, heat production, and locomotor activity were measured using the Oxymax system (Columbus Instruments).

The serum leptin concentration was measured using a mouse leptin ELISA kit (BioVender). Serum NEFA and glycerol levels were measured using an NEFA C-test (Wako) and free glycerol assay kit (BioVision), respectively. The serum GOT, GPT, and LDH levels were measured using a GOT/GPT test kit (Wako) and Cytotoxicity Detection Kit (LDH) (Roche), respectively. Isolation and cell fractionation of WAT were performed as described previously (7). Hepatic and muscle TG content was measured as described previously (50, 51).

### Immunoblotting.

Rabbit polyclonal anti-ALK7 Ab was generated as described previously (5). Goat anti–mouse IgG2a, Fc-γ–specific Ab (catalog 33416) and rabbit mAbs against ATGL (catalog 2439S, clone 30A4, RRID:AB_2167953), Smad3 (catalog 9523, clone C67H9, RRID:AB_2193182), phospho-Smad3 (Ser 423/425) (catalog 9520, clone C25A9, RRID:AB_2193207), and UCP-1 (catalog 14670S, clone D9D6X, RRID:AB_2687530) were purchased from Cell Signaling Technology. Mouse monoclonal anti–β-actin Ab (catalog A5316, clone AC-74, RRID:AB_476743) was purchased from MilliporeSigma. Rabbit polyclonal anti–β_3_ adrenergic receptor Ab (ab94506, RRID:AB_10863818) was purchased from Abcam. Rabbit monoclonal anti-GAPDH Ab (catalog M171-3, RRID:AB_1059773) and polyclonal anti–HA-tag Ab (catalog 561, RRID:AB_591839) were purchased from MBL. Immunoblotting was performed as described previously (7).

### Binding experiments in HEK293T cells.

HEK293T cells (ATCC) were cultured in high-glucose DMEM containing 10% FBS and 1% penicillin/streptomycin in 24-well plates. We transfected 90% confluent cells with 250 ng of pcDNA3-HAC or that harboring mouse ALK4, ALK5, or ALK7 cDNA using the Lipofectamine 3000 Reagent kit (Invitrogen) and Opti-MEM (Gibco). After culturing for 48 hours, the cells were incubated with ALK7 mAb (300 nM) and cultured for 30 minutes. After PBS washing, cell lysates were harvested.

### RNA preparation and gene expression analyses.

RNA was extracted using Sepasol-RNA I Super (Nacalai Tesque). Total RNA (1 μg) was reverse transcribed using oligo-(dT)12-18 primer and Superscript III (Invitrogen). Real-time PCR was performed as described previously (7) with TB Green premix Ex Taq (Takara Bio) using a C1000 Thermal Cycler (Bio-Rad). The results were normalized to 36B4 mRNA expression and then to values obtained from control samples. The primer sequences used are listed in Supplemental Table 1.

### Histological examination.

FFPE liver sections (2 to ~3 μm) were stained with H&E to visualize morphology or Sirius Red (catalog 196-16201; Wako) to assess collagen deposition. For F4/80 detection, liver sample slides were reacted with an F4/80 Ab (1:200 dilution; ab111101; Abcam; RRID:AB_10859466) at 4°C overnight followed by a secondary HRP polymer-conjugated goat anti–rabbit IgG Ab (1:2,000 dilution; ab214880; Abcam) for 60 minutes at room temperature and were developed with DAB (Dako). After counterstaining with hematoxylin, representative images were acquired using DP2-BSW software (Olympus).

### Measurement of FAO capacity in muscle.

FAO capacity in type I–dominant red muscle was measured as previously reported (52) with slight modifications. In brief, isolated soleus muscle was homogenized in distilled deionized water containing 250 mM sucrose, 1 mM EDTA, and 10 mM Tris-HCl, pH 7.4. Then, homogenates were incubated with [^14^C]-palmitic acid (0.2 μCi/mL; PerkinElmer) in 1 mL of incubation buffer (100 mM sucrose, 10 mM Tris-HCl, pH 7.4, 5 mM KH_2_PO_4_, 80 mM KCl, 1 mM MgCl_2_, 2 mM l-carnitine, 0.1 mM malate, 2 mM ATP, 0.05 mM coenzyme A, 1 mM dithiothreitol, 0.2 mM EDTA, and 0.3% fatty acid–free BSA dissolved in distilled deionized water) at 37°C for 2 hours. After stopping the oxidation by addition of 0.1 mL of 2 M HCl, filter paper soaked in 2 M NaOH was briefly placed into the upper section of the homogenates in the same tube, and the ^14^CO_2_ in the filter paper, a final product of oxidated [^14^C]-palmitic acid in muscle homogenates, was counted using a liquid scintillation counter.

### Ex vivo ATM and epiWAT, and primary adipocyte culture.

CD11c^–^ ATMs were isolated by FACS from epiWAT SVF as described previously (7) and cultured in 24-well plates in FBS-free DMEM containing 1% penicillin/streptomycin with or without reagents such as recombinant mouse IL-1β (1 or 10 ng/mL; Biolegend), S100A8 and S100A8/A9 heterodimer (both at 10 μg/mL; Biolegend), and LPS (1 μg/mL; Sigma-Aldrich). Wortmannin (100 nM; Sigma-Aldrich), the PI3K inhibitor, was added to the indicated well 10 minutes prior to addition of IL-1β. MCC950 (10 μM; Selleckchem), the NLRP3 inhibitor (22), was added to the indicated well 30 minutes prior to addition of S100A8/A9. After culturing for 24 hours, the supernatants and cell pellets were harvested and subjected to further analysis. The GDF3 protein concentration in the supernatant was measured using a mouse GDF3 ELISA Kit (Elabscience).

The ex vivo epiWAT culture was conducted as previously reported (53) with slight modifications. In brief, 0.5 g of isolated epiWAT explant was minced into small pieces (approximately 0.2 cm on a side) and cultured in DMEM containing 20% FBS and penicillin/streptomycin in the presence or absence of recombinant human (rh) GDF3 (400 ng/mL; R&D Systems) for 24 hours. To determine the level of S100A8/A9 protein in the epiWAT, 0.05 g of isolated epiWAT was homogenized using a plastic homogenizer in 500 μL of PBS containing protease inhibitor cocktail (Roche) and 0.5% Triton X-100, followed by 10 seconds of sonication. After centrifugation at 10,000*g* at 4°C for 20 minutes, the S100A8/A9 heterodimer concentrations in the supernatants were determined using a mouse S100A8/S100A9 heterodimer ELISA kit (R&D Systems). MCC950 (10 μM, 30 minutes), paquinimod (900 μg/mL, 10 minutes; Sigma-Aldrich), the S100A8/A9 inhibitor (54), FPS-ZM1 (30 μg/mL, 2 hours; Calbiochem), the RAGE antagonist (55), wortmannin (100 nM, 10 minutes), or ALK7 mAb (100 or 300 nM, 30 minutes) was added to the indicated well prior to addition of rhGDF3. After centrifugation at 50*g* at 4°C for 5 minutes, the epiWAT explants were removed. The S100A8/A9 protein levels in the lysates of 0.1 g of epiWAT were measured by ELISA as described above. The mRNA levels in SVF isolated from approximately 0.35 g of epiWAT were determined by real-time RT-PCR. The epiWAT-derived primary adipocytes were cultured at 37°C in 24-well plates in Krebs-Ringer HEPES buffer (20 mM HEPES, pH 7.4, 120 mM NaCl, 5 mM KCl, 2 mM CaCl_2_, 1 mM MgCl_2_, and 1 mM KH_2_PO_4_) containing 2 mmol/L glucose and 1% fatty acid–free BSA, as described previously (7). The adipocytes were incubated with rhGDF3 (400 ng/mL) for 30 minutes with or without ALK7 mAb (300 nM) pretreatment for 30 minutes.

### Statistics.

All quantitative data were expressed as the mean ± SD unless otherwise indicated. Data were analyzed using GraphPad Prism software. The *P* values were calculated using 2-tailed a *t* test or 1-way or 2-way ANOVA with a Tukey multiple-comparison test to determine significant differences between the group means. *P* < 0.05 was considered statistically significant.

### Study approval.

Animal experiments were performed in accordance with rules and regulations of and with the approval of the Animal Care and Experimentation Committee, Gunma University (approval no. 22-009).

## Author contributions

MZ and YB performed most experiments except hyperinsulinemic-euglycemic clamp, which was performed and supervised by OK and TK, respectively, and pancreatic islet studies, which were performed by HW. KO directly supervised both MZ and YB performing the experiments. TI designed the whole project and wrote the article on the basis of contributions from KO about the information of experimental procedures. The order of the 3 first authors was assigned by consideration of contributions of each author to the accomplishment of the work. Namely, although YB performed the initial experiments, MZ and KO expanded and completed the project, including the additional experiments in the revision process.

## Supplementary Material

Supplemental data

## Version 1 01/10/2023

In-Press Preview

## Version 2 02/22/2023

Electronic publication
