# Supplementary material for: Targeting activin receptor–like kinase 7 ameliorates adiposity and associated metabolic disorders
Source: JCI Insight. 2023 Feb 22;8(4):e161229. doi: 10.1172/jci.insight.161229 (PMC9977491; doi:10.1172/jci.insight.161229)
Supplement: Supplemental data [file jciinsight-8-161229-s167.pdf]

Supplemental Information

Supplemental Figures

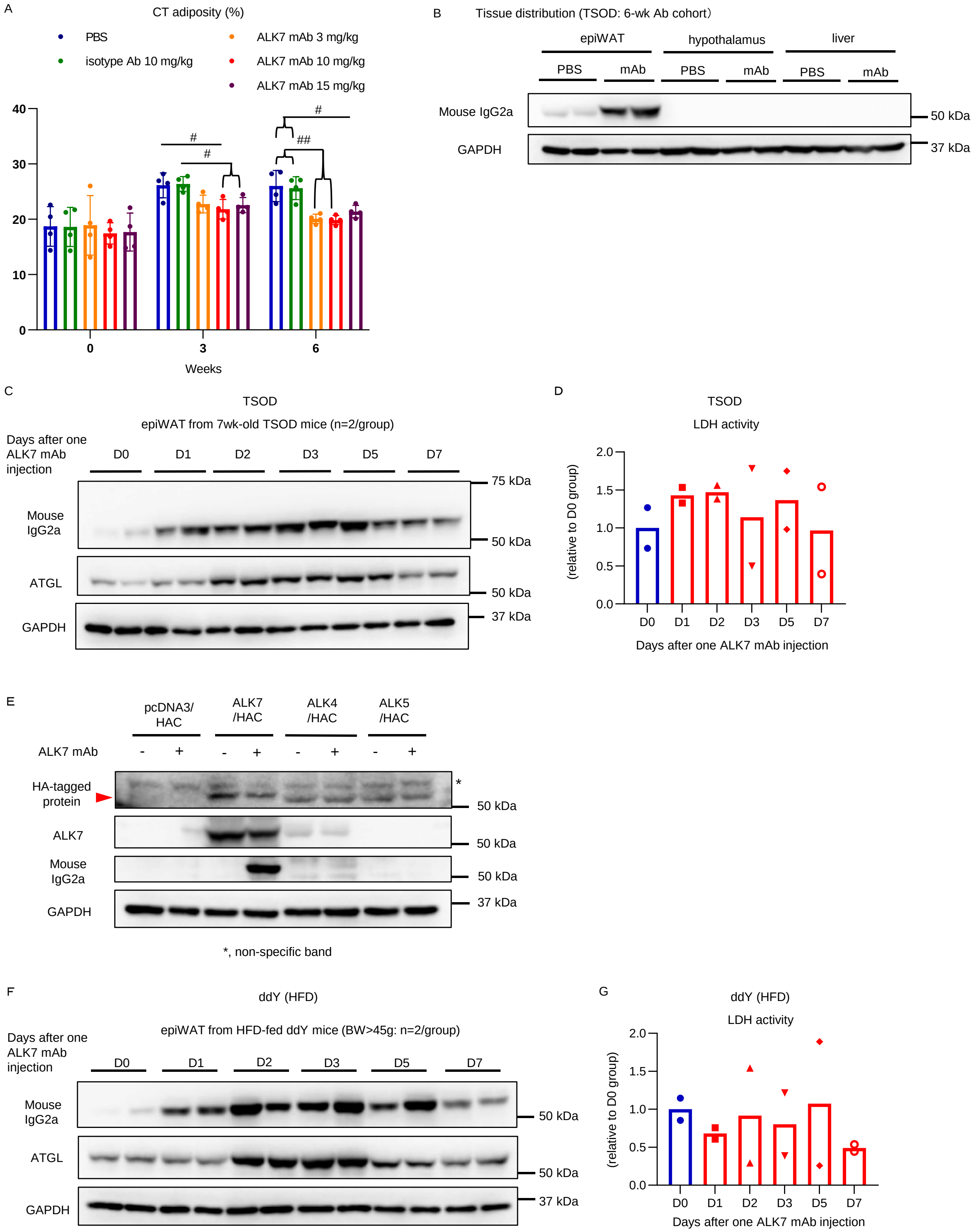

Supplemental Figure 1. Basic characterization of ALK7 mAb

A: TSOD mice were treated with ALK7 mAb (3,10, and 15 mg/kg), the isotype antibody (10 mg/kg), or PBS twice weekly for 6 weeks from 5 to 11 weeks of age. CT adiposity was examined at 0, 3, and 6 weeks after the treatment (*n* = 4 each). B: TSOD mice were treated with ALK7 mAb (10 mg/kg) or PBS for 6 weeks as described in A. The biodistribution of the injected antibody was examined in extracts of epiWAT, hypothalamus, and liver by immunoblotting with antibodies against mouse IgG2a and GAPDH (*n* = 2 each). C and D: TSOD mice at the age of 7 weeks were treated with ALK7 mAb (10 mg/kg) one time. epiWAT and serum samples were harvested on day 0 (without antibody injection), 1, 2, 3, 5, and 7. Protein levels of IgG2a-Fc, ATGL, and GAPDH in epiWAT (C) and LDH activity in serum (D) were measured (*n* = 2 each). E: HEK293T cells were transfected with 250 ng of pcDNA3-HAC or that containing mouse ALK4, ALK5, or ALK7 cDNA. Transfected cells were incubated with ALK7 mAb (300 nM) for 30 min and were washed by PBS twice before harvesting the cell lysates. The lysates were immunoblotted by the antibodies toward the indicated proteins (*n* = 3). * non-specific bands. F and G: HFD-fed ddY mice whose BW reached 45 g were treated with ALK7 mAb (10 mg/kg) one time. Protein levels of IgG2a-Fc, ATGL, and GAPDH in epiWAT (F) and LDH activity in serum (G) were measured as in C and D (*n* = 2 each). Shown are representative immunoblots of the indicated protein in the lysate. $^{\#}P < 0.05$, $^{\#\#}P < 0.01$; one-way ANOVA.

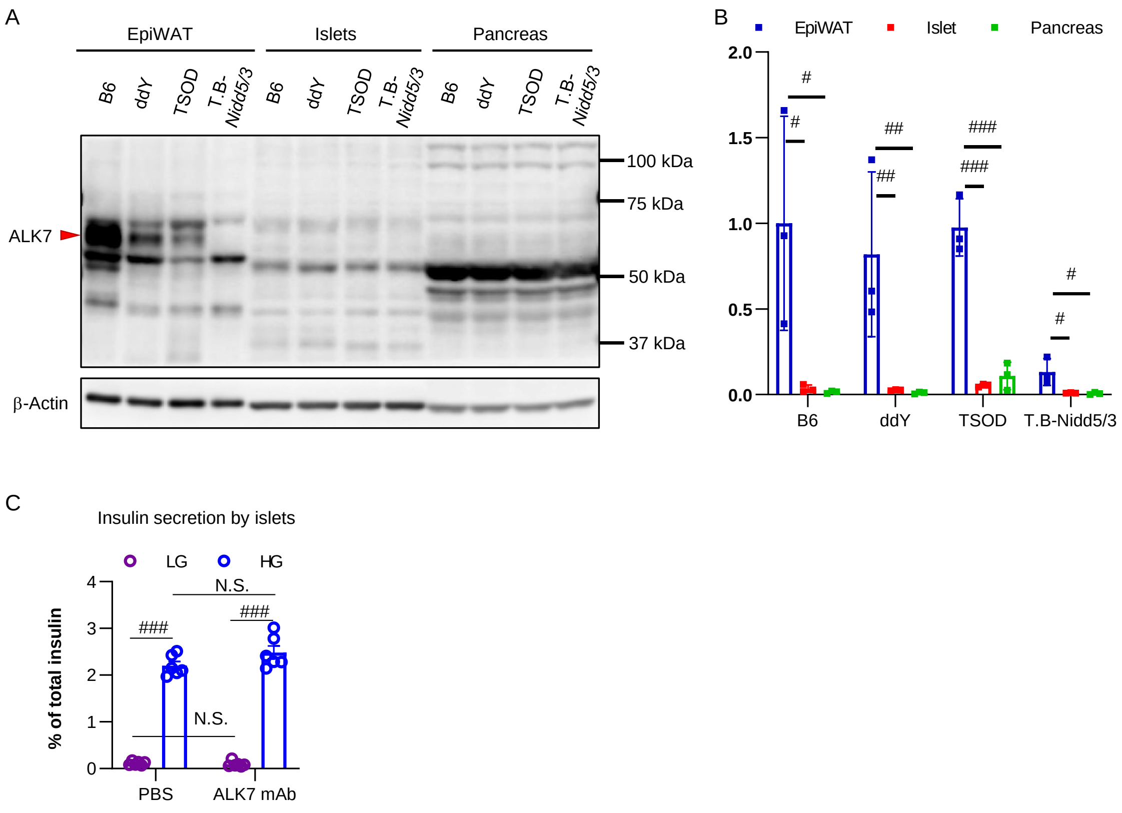

Supplemental Figure 2. ALK7 mAb treatment does not affect insulin secretion from pancreatic islets

A: ALK7 protein levels in extracts (10 µg) of epiWAT, islets, and pancreas from C57BL/6N (B6), ddY, TSOD, and T.B-*Nidd5*/3 mice were examined by immunoblots with polyclonal anti-ALK7 antibody. B: ALK7 mRNA levels in epiWAT, islets, and pancreas from C57BL/6N, ddY, TSOD, and T.B-*Nidd5*/3 mice were examined by real-time RT-PCR. Values are normalized to the average value of epiWAT from C57BL/B6N mice (n = 3 each). C: TSOD mice were treated with ALK7 mAb (10 mg/kg) or PBS for 16 weeks starting at 5 weeks of age, as described in Figure 1. Pancreatic islets isolated at 20 weeks of age were incubated at low glucose (LG, 2.8 mM) and high glucose (HG, 16.7 mM) concentrations for 30 min. Insulin levels secreted in the media and left in the cell extracts were measured, and their ratios are shown (n = 6 each). $^{\#}P < 0.05$, $^{\#\#}P < 0.01$, $^{\#\#\#}P < 0.001$, N.S. not significant; one-way ANOVA.

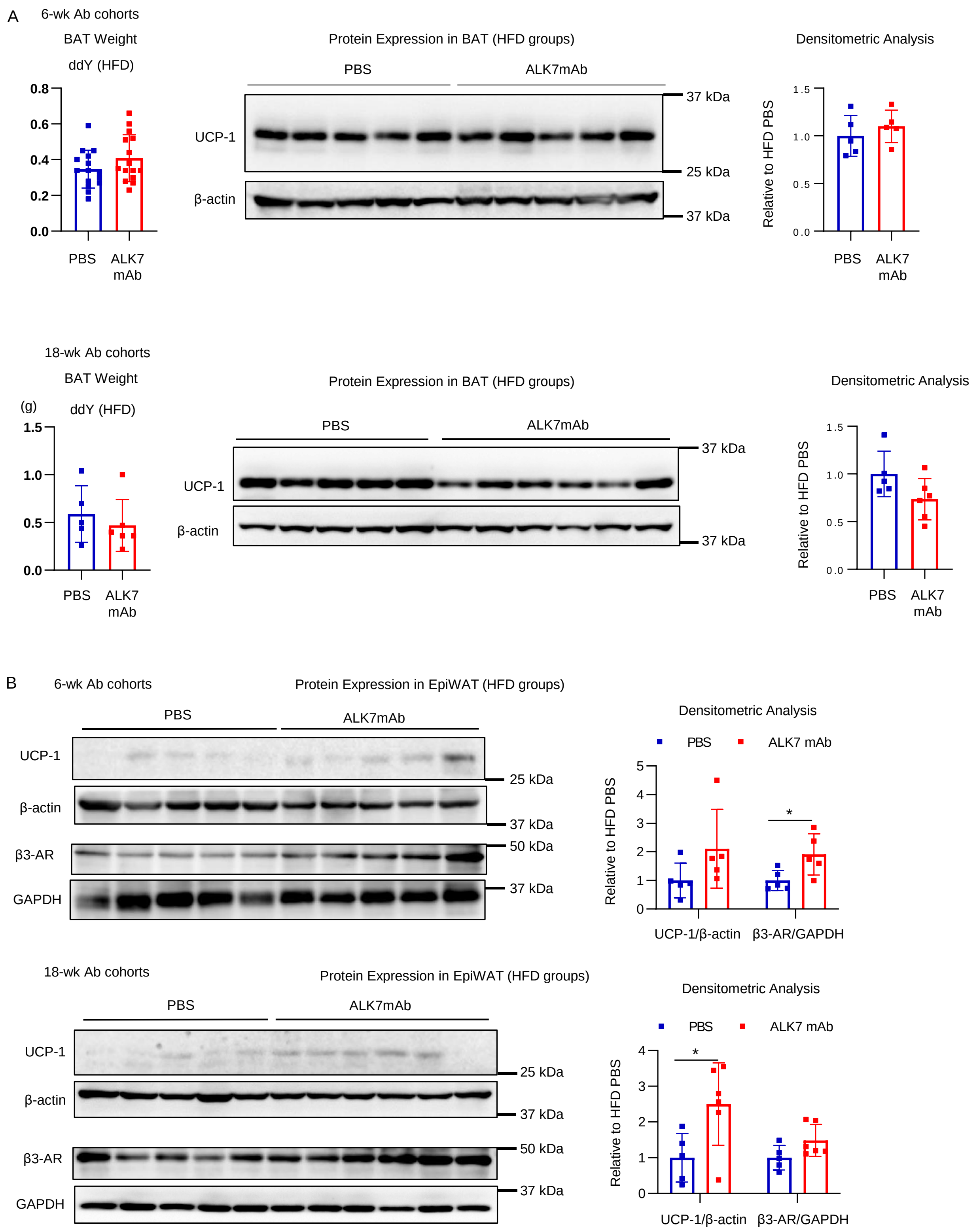
A
6-wk Ab cohorts
BAT Weight
ddY (HFD)
0.8
0.6
0.4
0.2
0.0
PBS
ALK7 mAb
Protein Expression in BAT (HFD groups)
PBS
ALK7mAb
UCP-1
β-actin
37 kDa
25 kDa
37 kDa
Densitometric Analysis
Relative to HFD PBS
1.5
1.0
0.5
0.0
PBS
ALK7 mAb
18-wk Ab cohorts
BAT Weight
(g)
ddY (HFD)
1.5
1.0
0.5
0.0
PBS
ALK7 mAb
Protein Expression in BAT (HFD groups)
PBS
ALK7mAb
UCP-1
β-actin
37 kDa
37 kDa
Densitometric Analysis
Relative to HFD PBS
1.5
1.0
0.5
0.0
PBS
ALK7 mAb
B
6-wk Ab cohorts
Protein Expression in EpiWAT (HFD groups)
PBS
ALK7mAb
UCP-1
β-actin
β3-AR
GAPDH
25 kDa
37 kDa
50 kDa
37 kDa
Densitometric Analysis
PBS
ALK7 mAb
Relative to HFD PBS
5
4
3
2
1
0
*
UCP-1/β-actin
β3-AR/GAPDH
18-wk Ab cohorts
Protein Expression in EpiWAT (HFD groups)
PBS
ALK7mAb
UCP-1
β-actin
β3-AR
GAPDH
25 kDa
37 kDa
50 kDa
37 kDa
Densitometric Analysis
PBS
ALK7 mAb
Relative to HFD PBS
4
3
2
1
0
*
UCP-1/β-actin
β3-AR/GAPDH

Supplemental Figure 3. ALK7 mAb treatment does not affect BAT weight or UCP1 protein expression, but tends to increase UCP1 and β3 adrenergic receptor protein expression in WAT

HFD-fed ddY mice were treated with ALK7 mAb (10 mg/kg) or PBS for 6 or 18 weeks, as described in Figure 2 (*n* = 5~6 per group). Shown are weight of interscapular BAT (A), immunoblots of indicated proteins in extracts (15 μg) of BAT (A) or epiWAT (B), and densitometric quantification of protein levels (A and B). β3-AR, β3 adrenergic receptor. $^{*}P < 0.05$; *t* test.

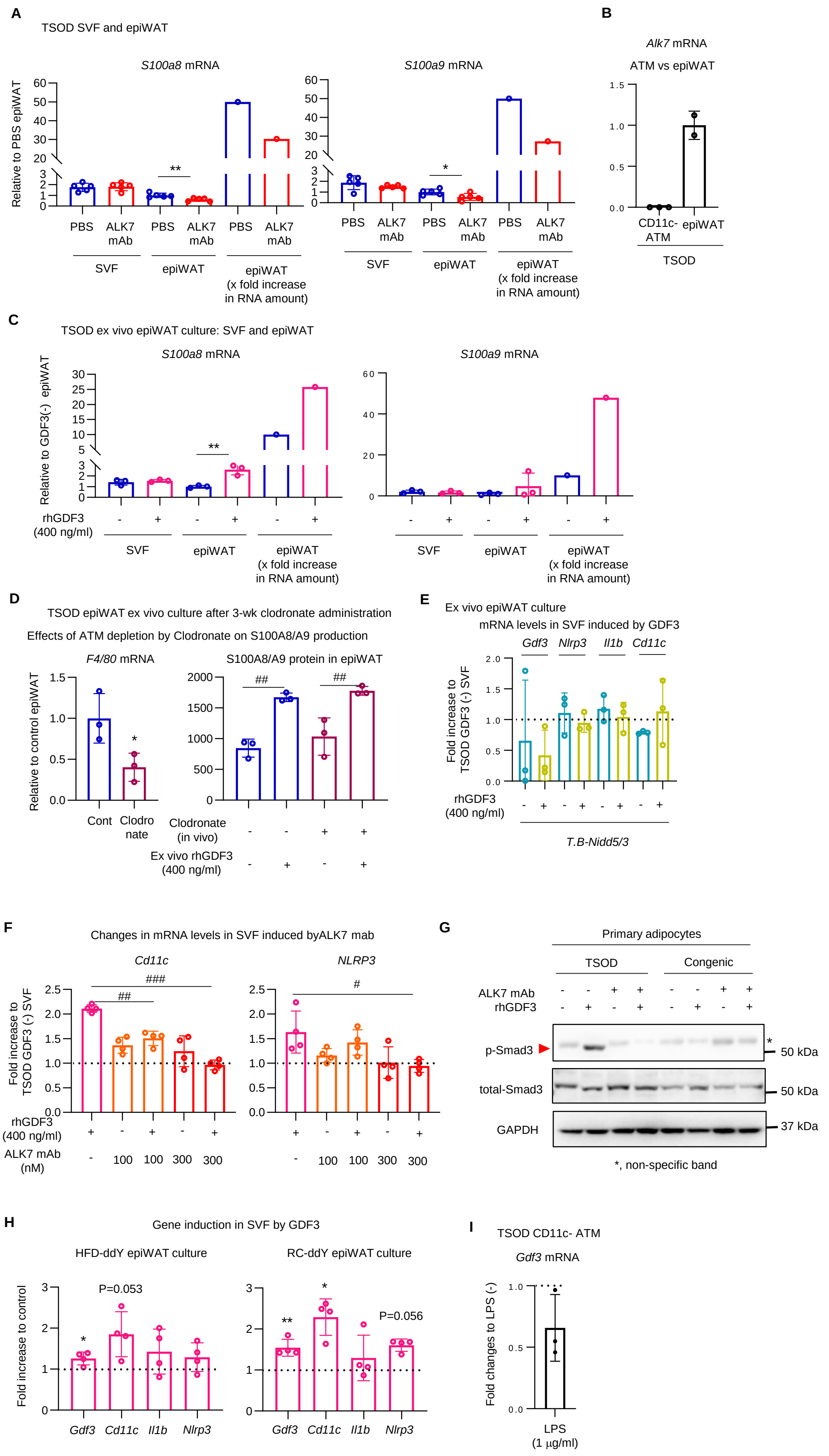
A
TSOD SVF and epiWAT
S100a8 mRNA
S100a9 mRNA
Relative to PBS epiWAT
PBS
ALK7 mAb
SVF
epiWAT
epiWAT (x fold increase in RNA amount)
B
Alk7 mRNA
ATM vs epiWAT
CD11c- ATM
epiWAT
TSOD
C
TSOD ex vivo epiWAT culture: SVF and epiWAT
S100a8 mRNA
S100a9 mRNA
Relative to GDF3(-) epiWAT
rhGDF3 (400 ng/ml)
SVF
epiWAT
epiWAT (x fold increase in RNA amount)
D
TSOD epiWAT ex vivo culture after 3-wk clodronate administration
Effects of ATM depletion by Clodronate on S100A8/A9 production
F4/80 mRNA
S100A8/A9 protein in epiWAT
Relative to control epiWAT
Cont
Clodronate
Clodronate (in vivo)
Ex vivo rhGDF3 (400 ng/ml)
E
Ex vivo epiWAT culture
mRNA levels in SVF induced by GDF3
Gdf3
Nlrp3
Il1b
Cd11c
Fold increase to TSOD GDF3 (-) SVF
rhGDF3 (400 ng/ml)
T.B-Nidd5/3
F
Changes in mRNA levels in SVF induced byALK7 mab
Cd11c
NLRP3
Fold increase to TSOD GDF3 (-) SVF
rhGDF3 (400 ng/ml)
ALK7 mAb (nM)
G
Primary adipocytes
TSOD
Congenic
ALK7 mAb
rhGDF3
p-Smad3
total-Smad3
GAPDH
50 kDa
50 kDa
37 kDa
*, non-specific band
H
Gene induction in SVF by GDF3
HFD-ddY epiWAT culture
RC-ddY epiWAT culture
Fold increase to control
P=0.053
P=0.056
Gdf3
Cd11c
Il1b
Nlrp3
I
TSOD CD11c- ATM
Gdf3 mRNA
Fold changes to LPS (-)
LPS (1 μg/ml)

Supplemental Figure 4. GDF3-ALK7 axis increases S100A8/A9 production in adipocytes

A: TSOD mice were treated with ALK7 mAb or PBS for 6 weeks, as described in Figure 1. S100A8 and S100A9 mRNA levels in epiWAT and SVF were determined by real-time RT-PCR (*n* = 5). Values were normalized to the averages of those in epiWAT obtained from PBS-treated mice. Considering that RNA amounts extracted from total epiWAT and SVF were on average ~100 μg and ~2 μg per 1g of epiWAT, respectively, the S100A8 and A9 mRNA levels in epiWAT were multiplied by 50 to compare the expression levels in SVF from the same weight of epiWAT (shown on the right side of each graph). Data for total epiWAT are identical to those in Figure 7C. B: ALK7 mRNA levels were measured in $CD11c^-$ ATM (*n* = 3) and epiWAT (*n* = 2) obtained from 7-week-old TSOD mice. C: EpiWAT from TSOD mice were cultured ex vivo, as described in Figure 7B. After a 24-h culture, mRNA levels of S100A8 and A9 in epiWAT were determined (*n* = 3). Based on the RNA levels ratio (12 μg in total epiWAT vs. 1.2 μg in SVF from 0.5 g epiWAT), the mRNA levels in epiWAT were multiplied by 10 for comparison (shown on the right side of each graph). Considering that the mRNA levels of S100A8 and S100A9 in total epiWAT are much higher than those of SVF obtained from the same amount of epiWAT (A, C), that ALK7 is not expressed in ATMs (B), and that the mRNA levels of S100A8 and S100A9 are not induced in SVF by GDF3 administration to epiWAT (C), the adipocytes appear to be the main source of GDF3-induced S100A8/A9 production in epiWAT. D: TSOD mice at the age of 5 weeks received intraperitoneal injections of clodronate (110 mg/kg) encapsulated in liposomes, or liposomes alone as a control, twice per week for 3 weeks. F4/80 mRNA levels were measured by real-time RT-PCR in isolated epiWAT. Isolated epiWAT (0.5 g) were also cultured with or without rhGDF3 (400 ng/ml) for 24 h as described in Figure 8A and then, protein levels of S100A8/A9 in epiWAT were determined by ELISA. (*n* = 3). E-H:

EpiWAT (0.5 g) isolated from ALK7-deficienct T.B-*Nidd5/3* mice (E), ALK7-intact TSOD mice (F), or HFD-fed or RC-fed ddY mice (H) were incubated with or without rhGDF3 (400 ng/ml) ex vivo, and gene expression analysis was conducted (E, n = 3; F and H, n = 4), as described in Fig. 7B. In G, primary adipocytes isolated from epiWAT of 7-week-old TSOD or T.B-*Nidd5*/3 mice were cultured with or without rhGDF3 (400 ng/ml) for 30 min, and then, phosphorylation levels of Smad3 in cell lysates were determined by immunoblotting. In F and G, ALK7 mAb (300 nM) was added onto some wells 30 min prior to the addition of GDF3. Note that GDF3 signaling was almost completely blocked in ALK7-deficienct T.B-*Nidd5/3* mice (E, G), or by treatment of ALK7 mAb (F, G). I: $CD11c^-$ ATMs isolated from epiWAT of 7- to 8-week-old TSOD mice were cultured in the presence or absence of LPS at 1 μg/ml for 24 h. Then, the GDF3 mRNA levels in the cells were examined (n = 3). The RNA level in LPS-treated ATM was normalized to that in ATM without LPS in each experiment. Note that TLR4 signaling alone is not sufficient for induction of GDF3 in ATMs. $^{*}P < 0.05$, $^{**}P < 0.01$; t test. $^{\#}P < 0.05$, $^{\#\#}P < 0.01$, $^{\#\#\#}P < 0.001$; one-way ANOVA.

Supplemental Table 1. The primer sequences used in the present study

Gene (mouse)	Primer	Sequence
36B4	Forward	GGCCCTGCACTCTCGCTTTC
	Reverse	TGCCAGGACGCGCTTGT
PPARa	Forward	CTGAGACCCTCGGGGAAC
	Reverse	AAACGTCAGTTCACAGGGAAG
PPARd	Forward	ATGGGGGACCAGAACACAC
	Reverse	GGAGGAATTCTGGGAGAGGT
CPT1b	Forward	TGCCTTTACATCGTCTCCAA
	Reverse	GGCTCCAGGGTTCAGAAAGT
PGC-1a	Forward	GAAAGGGCCAAACAGAGAGA
	Reverse	GTAAATCACACGGCGCTCTT
Ucp2	Forward	AGCCTGAGACCTCAAAGCAG
	Reverse	CCTTCAATCGGCAAGACG
Ucp3	Forward	TACCCAACCTTGGCTAGACG
	Reverse	GTCCGAGGAGAGAGCTTGC
ACOX-1	Forward	GCCCAACTGTGACTTCCATC
	Reverse	GCCAGGACTATCGCATGATT
LCAD	Forward	TGGGGACTTGCTCTCAACA
	Reverse	GGCCTGTGCAATTGGAGTA
MCAD	Forward	TGTCGAACACAACACTCGAAA
	Reverse	CTGCTGTTCCGTCAACTCAA
Nlrp3	Forward	ACTTGCAGAAGCTGGGGTTG
	Reverse	AGTTTACAGTCCGGGTGCAG
Il1b	Forward	GGAGAACCAAGCAACGACAAAATA
	Reverse	TGGGGAACTCTGCAGACTCAAAC
Gdf3	Forward	CGCAGGACTTATGCTACGTG
	Reverse	AGACAGGAGCCATCTTGGAA
S100a8	Forward	GACAATGCAATTAACTTCGAGGAG
	Reverse	TGTGGCTGTCTTTGTGAGATGC
S100a9	Forward	CAGCATAACCACCATCATCG
	Reverse	GTCCTGGTTTGTGTCCAGGT
Alk7	Forward	ATCCTCGGTTTCATCGCAGC

	Reverse	ACCATTCCAGCCACAGTCAC
Cd11c	Forward	CTGGATAGCCTTTCTTCTGCTG
	Reverse	GCACACTGTGTCCGAACTCA
Col1a1	Forward	GAGAGAGCATGACCGATGGATT
	Reverse	TGTAGGCTACGCTGTTCTTGCA
Col1a2	Forward	GCAGGGTTCCAACGATGTTG
	Reverse	GCAGCCATCGACTAGGACAGA
Acta2	Forward	ACTGGGACGACATGGAAAAG
	Reverse	GTTCAGTGGTGCCTCTGTCA
Mmp13	Forward	AGAAGTGTGACCCAGCCCTA
	Reverse	GCGCAAGAAGAATCTGTCTTT
IL-1a	Forward	CGCTTGAGTCGGCAAAGAAA
	Reverse	TGATACTGTCACCCGGCTCT
TGFb1	Forward	TGGAGCAACATGTGGAACTC
	Reverse	CAGCAGCCGGTTACCAAG
TNFa	Forward	GCCTCTTCTCATTCCTGCTTG
	Reverse	CTGATGAGAGGGAGGCCATT
MCP1	Forward	AGGTCCCTGTCATGCTTCTG
	Reverse	GCTGCTGGTGATCCTCTTGT
F4/80	Forward	GGAGGACTTCTCCAAGCCTATT
	Reverse	AGGCCTCTCAGACTTCTGCTT
